# Tumour microenvironment landscape and immunotherapy response in bladder cancer decoded by stromal MOXD1 based on copper-related genes signature

**DOI:** 10.3389/fonc.2022.1081091

**Published:** 2022-12-22

**Authors:** Wenhao Wang, Shan Hua, Jianying Li, Jing Zhao, Yu Zhang, Juntao Jiang, Bangmin Han

**Affiliations:** Department of Urology, Shanghai General Hospital, Shanghai Jiao Tong University School of Medicine, Shanghai, China

**Keywords:** bladder cancer, copper, signature, tumor-infiltrating immune cell, immunotherapy, fibroblast

## Abstract

**Introduction:**

We aimed to develop a copper-related gene (CRG) signature that can be used to evaluate prognosis and guide therapeutic management in bladder cancer patients.

**Methods:**

The raw transcriptome profiles and clinical data of 405 bladder samples were downloaded from The Cancer Genome Atlas (TCGA) database, and differentially expressed copper-related genes were identifified using the Molecular Signatures Database (MSigDB) database and univariate and multivariate Cox regression analysis. A multigene prognostic signature based on 14 CRGs was developed by least absolute shrinkage and selection operation (LASSO) analysis in the TCGA cohort and validated in the Gene Expression Omnibus (GEO) cohort. Multiple analyses were then conducted in which the nomograms, clinicopathological features, immune-related cell infifiltration characteristics, and therapy responses of the high- and low-risk score groups were compared.

**Results:**

A 14 CRGs signature was constructed and used to classify patients into high-risk and low-risk groups. Compared to patients classifified as high-risk, low-risk patients in both the TCGA cohort and the GEO cohort had better overall survival. Patients in high-risk groups had more aggressive clinical features, immunologically “cold” infifiltrating characteristics, and experienced lower therapeutic effificacy. We identifified a CRG signature of bladder cancer and validated it using unsupervised clustering analysis. Monooxygenase DBH-like 1 (MOXD1) was further identifified, and its potential for evaluating the tumor immune microenvironment and predicting the immunotherapy response was explored.

**Discussion:**

These results suggest a novel research direction for precision therapy of bladder cancer and demonstrate that copper-related genes can play a promising role in predicting prognosis and may serve as therapeutic targets for bladder cancer.

## 1 Introduction

Bladder urothelial carcinoma (BLCA) is the most common malignant tumor of the urinary system. It is estimated that 500,000 new cases of BLCA and 200,000 resulting deaths occur annually worldwide, and the disease is responsible for over 80,000 new cases and 17,000 deaths per year in the United States alone (1, 2). BLCA is characterized by a high recurrence rate and a proneness to metastasis, and the 5-year survival rate is generally <50% (3). Chemotherapy, surgery, and immunotherapy are currently the most effective approaches to the treatment of BLCA, but these approaches have limitations. Molecularly targeted therapies have emerged as revolutionary cancer treatments that can increase the survival time of patients (4). Nevertheless, widely accepted prognostic biomarkers for BLCA still do not exist, and there is a serious unmet need for the identification of reliable biomarkers that can be used to determine risk and devise personalized treatment regimens for individual BLCA patients.

Cuproptosis was first identified in March 2022 as a form of cell death characterized by mitochondrial respiration regulated in a copper-dependent manner. Cells with higher mitochondrial respiration activity display increased sensitivity to copper ionophores, which are copper-binding small molecules that transfer copper from extracellular to intracellular sites (5, 6). Generally, intracellular copper concentrations are maintained at very low levels by active homeostatic mechanisms. However, copper imbalance affects inflammation, organ development, lipid metabolism and even sensitivity to chemotherapeutics (7). The accumulated intracellular copper can induce cell death, and this process can be reversed in hundreds of cell lines by binding of copper to molecules present in the cells (8, 9). Cancer-related metabolic reprogramming, including altered fatty acid metabolism and glucose metabolism, has profound effects on tumorigenesis, and copper has been reported to play essential regulatory roles in many metabolic processes (10–12). Indeed, before the identification of cuproptosis, disordered copper metabolism was shown to play a role in cancer occurrence and progression. A recent study of triple-negative breast cancer reported that copper-enriched SOX2/OCT4+ cells showed much higher sensitivity than other cells to copper depletion and suggested that metabolic reprogramming of a select population of SOX2/OCT4+ metastatic cells in a way that leads to copper depletion could be a novel antimetastatic therapy (13). Another study suggested that inhibition of the copper-trafficking proteins Atox1 and CCS could disrupt cellular copper transport and thereby inhibit the proliferation of cancer cells as well as attenuate tumor growth (14). Growing tumors employ ATP7A/B, a Golgi-localized copper-transporting ATPase that functions in the maintenance of copper homeostasis, to maintain the concentration of copper needed for the activity of oncogenic enzymes such as LOX and LOX-like proteins (15). Many prior studies have suggested that copper-related small molecules that regulate copper transport and metabolism play a critical role in tumor initiation, progression, metastasis, and therapy.

In this study, genomic data obtained from 405 BLCA samples were comprehensively analyzed, the copper metabolism pattern reflected by the data was evaluated, and a copper prognostic risk score signature was developed. The prognostic risk signature, which is based on the patients’ clinical information, can not only independently predict survival outcome but also effectively identify and distinguish groups of patients who are resistant to chemotherapeutic drugs and targeted drugs. The relationship between the prognostic risk signature and tumor immune infiltration characteristics was also explored, and the bladder cancer patients were grouped according to their immune subtypes. Fourteen prognostic genes were identified, and MOXD1 was further investigated to explore its correlation with the immune landscape. The prognostic risk signature developed in this study confirms the important role of copper in shaping the individual tumor immune microenvironment and distinguishes populations of patients who have different responses to immune checkpoint inhibitors (ICIs). These conclusions provide novel perspectives for combining ionic therapy with immunotherapy for patients with bladder cancer.

## 2 Results

### 2.1 Difference analysis and construction of a prognostic risk score signature in the training set

**Figure 1:**
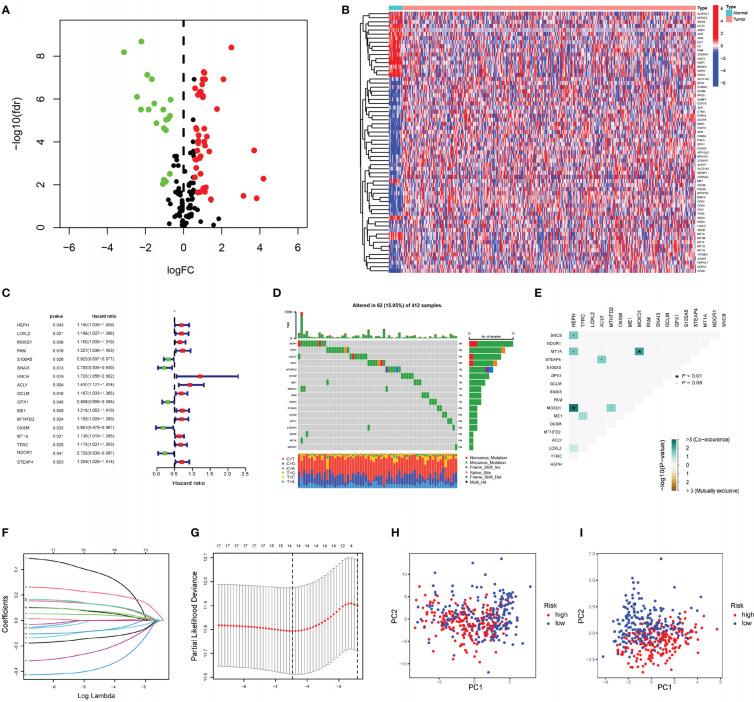
Construction of prognostic risk score signature **(A)** The volcano plot of 64 copper-related genes with significant difference (upregulated genes are marked in red; downregulated genes are marked in green). **(B)** The heatmap of differentially expressed copper-related gens in the normal and cancer tissue samples. **(C)** Forrest plot of 17 copper-related genes associated with prognosis. **(D)** The somatic mutation of 17 copper-related genes in 412 BLCA patients in TCGA cohort. **(E)** The analysis of mutation cooccurrence and exclusion for 17 prognostic copper-related genes. Cooccurrence, green; exclusion, brown. **(F)** The coefficients in LASSO Cox regression analysis of the 17 prognostic copper-related genes. **(G)** Identification of genes for construction of prognostic risk score signature. **(H)** Principal component analysis based on all copper-related genes in TCGA cohort. **(I)** Principal component analysis based on 14 prognostic signature copper-related genes in TCGA cohort. High-risk patients are represented by red group, and low-risk patients are represented by blue group.

Transcriptome profiling data for bladder tissue, based on the BLCA project reported in the TCGA database, which includes normal and cancer tissue samples, was conducted to analyze gene expression levels. The CRGs downloaded from MigSDB (https://www.gsea-msigdb.org/gsea/msigdb) were extracted (**Table S1**), and the differences were analyzed. In this cohort, 64 genes expressed in cancer tissue were selected according to a false discovery rate (FDR) < 0.05; 44 of these were upregulated, and 20 were downregulated. The differentially expressed CRGs are shown in **Figures 1A, B**. The expression profile data of differentially expressed CRGs obtained from the TCGA cohort in GSE13507 were extracted for validation (**Table S2**). Univariate Cox regression analysis was conducted on 64 differentially expressed CRGs comprising the training set in the TCGA cohort. Finally, 17 prognosis-related genes with p values <0.05 were identified (**Figure 1C**). Combined with the raw somatic mutation data obtained from the TCGA database, the mutation characteristics of these 17 CRGs were also analyzed and summarized. **Figure 1D** shows that somatic mutations in these 17 CRGs occurred in 62 of 412 bladder cancer patients, a frequency of 15.05%. Among these genes, HEPH had the highest mutation frequency, while STEAP4 was relatively stable with no somatic mutations. According to comutation analysis, HEPH and SNCB, HEPH and MTIA, and MOXD1 and HEPH exhibited a mutation cooccurrence relationship (**Figure 1E**). To construct a prognostic risk score signature, the candidate genes were narrowed to 14 genes (HEPH, LOXL2, MOXD1, S100A5, SNAI3, SNCB, ACLY, GCLM, GPX1, OXSM, MT1A, TFRC, NDOR1, and STEAP4) by LASSO Cox regression analysis (**Figures 1F, G**). The risk scores associated with the bladder cancer samples were calculated using the following formula: risk score = (0.0649294946722208)×HEPH + (0.00112844967059073)×LOXL2 + (0.0442203686581324)×MOXD1 + (-0.0793932777256413)×S100A5 + (-0.233746730976349)×SNAI3 + (0.3680400891395)×SNCB + (0.21480248315269)×ACLY + (0.123239938118587)×GCLM + (-0.0771053814083238)×GPX1 + (-0.133608314765734)×OXSM + (0.124543005735702)×MT1A + (0.0715649943387494)×TFRC + (-0.372743923494837) ×NDOR1 + (0.125154125013336) × STEAP4, as shown in **Table S2**.

Principal component analysis (PCA) demonstrated that the BLCA samples can be distinguished by the prognostic risk score signature (**Figures 1H, I**).

### 2.2 Correlation between risk score and clinical characteristics

**Figure 2:**
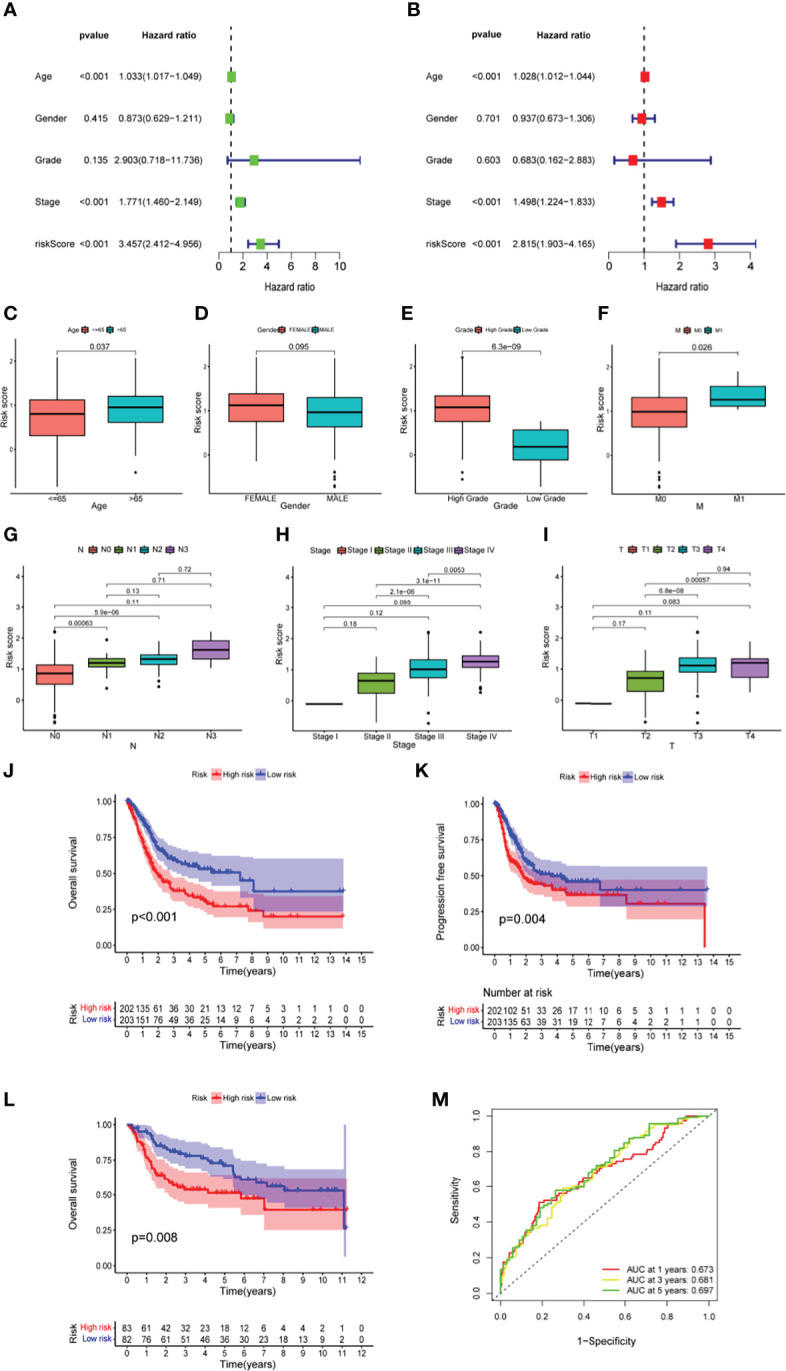
The predictive power of prognostic risk score signature in clinical characteristics among BLCA patients **(A, B)** The forest plot of the univariate and multivariate Cox regression analysis in TCGA cohort. **(C-I)** The relationship of risk score and clinical characteristics, including age **(C)**, sex **(D)**, tumour grade **(E)**, distal metastasis **(F)**, lymphoid metastasis **(G)**, TNM stage **(H)**, and tumor invasion **(I)**. **(J–L)** The comparison of overall survival (OS) between low- and high-risk score groups in the training set **(J)** and the test set **(L)**, and the progression-free survival (PFS) in training set **(K)**. **(M)** The prediction accuracy of the risk score measured by ROC curves at 1, 3, 5 years in the training set. The area under the curve (AUC) is 0.673, 0.681, 0.697 respectively.

Taking the median value of the risk score in the training set above as the cutoff value, a total of 405 ranked samples were divided into low-risk (n = 203) and high-risk (n = 202) groups. In univariate and multivariate Cox analyses, the indicators related to overall survival (OS) included not only risk score but also age and pathological stage, indicating that they can be used as independent prognostic indicators (**Figures 2A, B**). Furthermore, after redistributing the above samples according to the risk score, it was found that there was a significant correlation between risk score and changes in clinical indicators other than sex and age (**Figures 2C, D**), including stage, grade and American Joint Committee on Cancer (AJCC-TNM) criteria. Higher risk scores were associated with high-grade tumors (p < 0.001; **Figure 2E**). The risk score also had a positive correlation with AJCC-M (distal metastasis) (p = 0.026; **Figure 2F**) and AJCC-N (lymphoid metastasis) (p <0.01; **Figure 2G**). Among Stage II, Stage III and Stage IV patients, higher risk scores were associated with more advanced stage (p < 0.05; **Figure 2H**). Higher risk scores also correlated with higher AJCC-T (tumor invasion) stage in T2, T3, and T4 (**Figure 2I**). Compared to samples with high risk in the TCGA cohort, samples with low risk in the TCGA cohort had better OS (p <0.001; **Figure 2J**) and longer progression-free survival (PFS) (p = 0.004; **Figure 2K**). Among the samples in the test dataset from the Gene Expression Omnibus (GEO), GSE13507 was divided into low-risk (n = 82) and high-risk (n = 83) groups using the above-determined cutoff value, and the survival curves of these two groups were plotted (p = 0.008; **Figure 2L**). The risk scores and risk statuses of each sample are listed in Table S2. The prognostic factor-associated receiver operating characteristic (ROC) curves were plotted, and the area under the ROC curves (AUC) showed that the risk score was highly predictive of survival at 1 (AUC = 0.673), 3 (AUC = 0.681), and 5 (AUC = 0.697) years (**Figure 2M**). Overall, the low-risk samples had superior prognoses compared to those of the high-risk samples, suggesting that the prognostic risk score signature has a robust ability to predict OS in BLCA patients.

### 2.3 Construction of a nomogram for predicting prognosis

**Figure 3:**
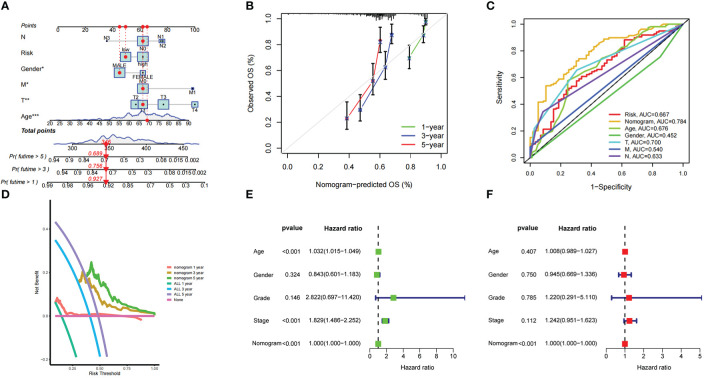
The predictive power of a nomogram incorporated with risk score and clinical features in overall survival of patients **(A)** Nomogram predicting overall survival of patients in training set. **(B)** The calibration plots of the nomogram at 1, 3, 5 years. The x coordinate value represents the nomogram-predicted survival, and the y coordinate value represents observed overall survival. **(C)** ROC curves for risk score, nomogram and clinical characteristics. **(D)** Decision analysis curve of the nomogram in the 1-year, 3-year, and 5-year. **(E, F)** The forest plot of the nomogram in univariate Cox and multivariate Cox regression analysis.

A nomogram for predicting OS in BLCA samples was constructed by integrating age, sex, stage, AJCC TNM and risk score (**Figure 3A**). As shown by the calibration curves for survival at 1, 3, and 5 years (**Figure 3B**), the nomogram accurately predicted OS in BCLA patients. As the AUC illustrates, compared with other indicators such as age (AUC = 0.676) and prognostic risk score signature (AUC = 0.667), the nomogram (AUC = 0.784; **Figure 3C**) had more promising prognostic value. Decision curve analysis showed that the nomogram had good prediction performance at 1, 3 and 5 years, especially at 3 and 5 years (**Figure 3D**). Although age and stage, which were related to overall survival in the univariate Cox analysis, had no correlation with overall survival in the multivariate Cox analysis, the nomogram always maintained good utility in predicting overall survival (**Figures 3E, F**).

### 2.4 Validation of the copper-related prognosis signature for bladder cancer by clustering analysis

**Figure 4:**
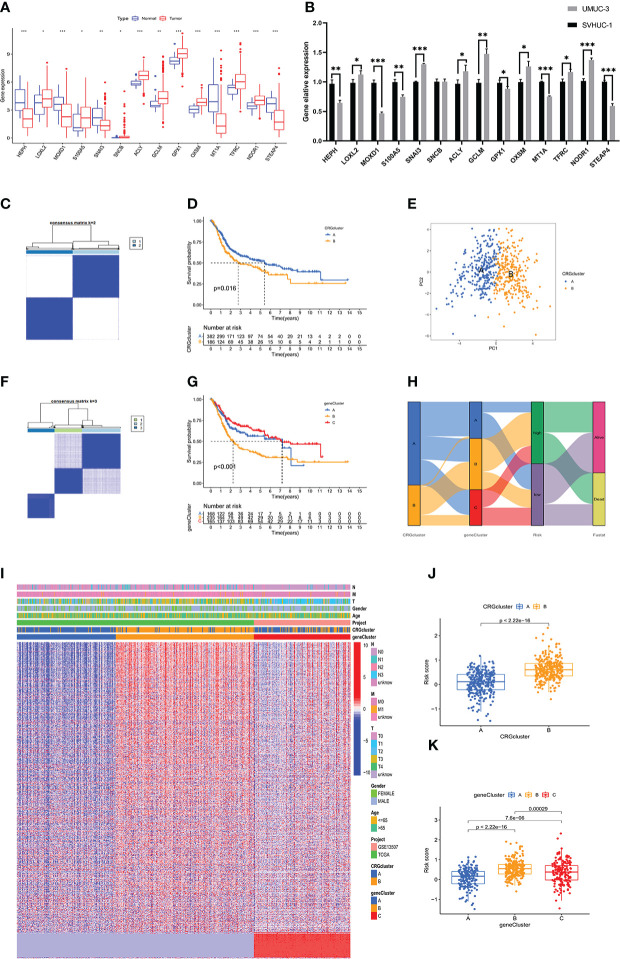
Validation of the copper-related prognosis signature for BLCA by clustering analysis **(A)** The expression level of 17 copper-related genes between the normal and tumour samples in the TCGA-BLCA dataset. **(B)** The mRNA level of 17 copper-related genes between the SVHUC-1 and UMUC3 cells. The results are presented as the mean ± SD of three independent experiments (*p < 0.05, **p < 0.01, ***p < 0.001). **(C, F)** The unsupervised clustering plots of TCGA-BLCA expression profile based on the prognosis related CRGs and all the prognosis related genes, respectively for two CRGclusters and three geneclusters. **(D, G)** Kaplan–Meier analysis of the OS in CRGclusters and geneclusters. **(E)** Principal component analysis of TCGA-BLCA samples through CRGclusters. **(H)** The alluvial diagram of copper-related prognosis signature risk scores, CRGclusters, geneclusters and clinical outcomes. **(I)** The heatmap of clinical features of the CRGclusters and geneclusters in BLCA samples. **(J, K)** The boxplot plots demonstrated the risk scores of CRGclusters and geneclusters.

First, we analyzed the differences in expression of 14 candidate genes in tumor and normal tissues based on the data in the TCGA-BLCA dataset. The data were screened using the prognostic model of copper-related genes (**Figure 4A**). qPCR was performed to validate the results in normal urothelial cells and bladder cancer cells *in vitro* (**Figure 4B**). Unsupervised clustering was then used to analyze the TCGA-BLCA expression profile based on the prognosis-related CRGs and on all the prognosis-related genes for two CRG clusters (A, B) (**Figure 4C**) and three gene clusters (A-C) (**Figure 4F**). Survival analysis showed that two types of clustering methods effectively predicted the OS of patients with bladder cancer (p value < 0.001) (**Figures 4D, G**). Principal component analysis showed that CRG clusters effectively distinguished bladder cancer samples (**Figure 4E**). The heatmap obtained after clustering is shown in **Figure 4I**. The alluvial diagram (**Figure 4H**) shows the distribution of the risk scores in the above copper-related prognostic models among different clusters and their correlation with clinical outcomes. The risk scores for the CRG clusters and gene clusters are also shown in boxplots (**Figures 4J, K**). In CRG clusters, Cluster B had a higher risk score than Cluster A, while in gene clusters, Cluster B had the highest risk score.

### 2.5 Immune-related features and response to immunotherapy in the low- and high-risk score groups

**Figure 5:**
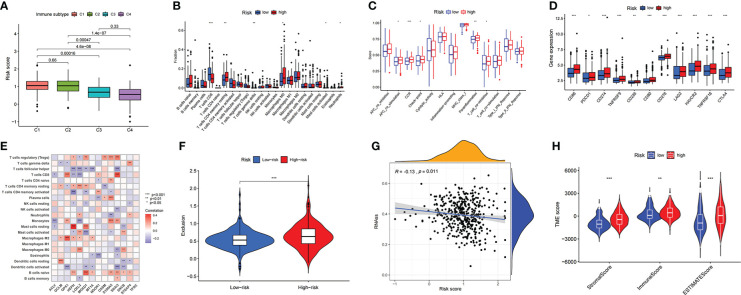
The description of immune-related characteristics in the BLCA samples based on copper prognostic signature **(A)** The relationship between risk score and immune subtype in the BLCA samples. **(B)** The immune cell infiltration difference between high- and low- risk score groups. **(C)** The difference of biological immune-related functions in high- and low- risk score groups. **(D)** The difference of ICIs-related molecules expressions in high- and low- risk score groups. **(E)** The correlation results of the expression of 14 prognostic genes and the numbers of various immune cells in the TCGA-BLCA cohort. **(F)** The prediction of different therapeutic responses to ICIs in BLCA cohort under the prognostic risk score signature. **(G)** The correlation between risk score of the CRG prognostic model and the presence of tumor stem cells. **(H)** The correlation between risk score of the CRG prognostic model and the tumor microenvironment score categories StromalScore, ImmuneScore, and EstimateScore.

A total of 411 BLCA samples were divided according to recently defined immune subtype categories; the samples were grouped into the wound-healing C1 immune subtype, the IFN-γ dominant C2 immune subtype, the inflammatory C3 immune subtype, and the lymphocyte-depleted C4 immune subtype. The relationship between subtype group and risk score calculated based on the prognostic signature described above is plotted in **Figure 5A**. The plot shows that the risk scores of bladder cancer patients with the C1 immune subtype are higher than those of patients with other immune subtypes. An analysis of the immune microenvironment and immune cell infiltration in patients with bladder cancer is shown in Table S3. The high-risk group showed remarkably poor immune-promoting cell population infiltration, including poor infiltration by CD8 T cells and activated dendritic cells (aDCs). Consistent with the OS advantage observed in the low-risk group compared to the high-risk group, M2 macrophages were inhibited in the low-risk group. CD8 T cells and T helper (Th) 17 cells were associated with poor survival outcomes in the high-risk group (**Figure 5B**). Correlation analysis of the expression of 14 prognostic genes and the numbers of various immune cells was also performed in the TCGA-BLCA cohort; the results are shown in **Figure 5E**. The analysis of immune function indicated that APC costimulation function, checkpoint function, and T-cell coinhibitory function were elevated in the high-risk group, demonstrating that immune-suppressed patients can respond to immunotherapy (**Figure 5C**). Although immunotherapy through administration of immune checkpoint inhibitors brings hope to cancer therapy, many cancer patients respond poorly to immune checkpoint inhibitors. Our investigation of the use of the prognostic risk score signature to distinguish BLCA patients with different responses to ICIs demonstrated that the expression levels of several significant immune checkpoint target molecules were higher in the high-risk group (**Figure 5D**) and that patients in the high-risk group displayed poorer therapeutic responses to ICIs, suggesting that quantification of the copper-related prognostic risk score signature is a promising predictor for indicating the therapeutic response to immunotherapy (**Figure 5F**). In addition, based on the treatment status and therapeutic response characteristics of bladder cancer patients receiving immunotherapy in the Tumor Immune Dysfunction and Exclusion (TIDE) database, we further found that bladder cancer patients in the high-risk group had higher scores for immune exclusion (**Figure S3C**), CD274 expression level, and MDSC grade than those in the low-risk group.

In addition to the above-described exploration of the relationship between risk scores and tumor immune characteristics, the differences between the high- and low-risk groups in tumor microenvironment were further investigated. This included confirming that the risk score of the CRG prognostic model was negatively correlated with the presence of tumor stem cells (**Figure 5G**); this demonstrated that the carcinogenic mechanism of CRGs in BLCA was not associated with mRNA expression-based stemness scores (RNAss). It was also confirmed that the high-risk group had higher scores in the tumor microenvironment score categories StromalScore, ImmuneScore, and EstimateScore (**Figure 5H**).

### 2.6 Gene set variation analysis and response to chemotherapy and to targeted therapy

**Figure 6:**
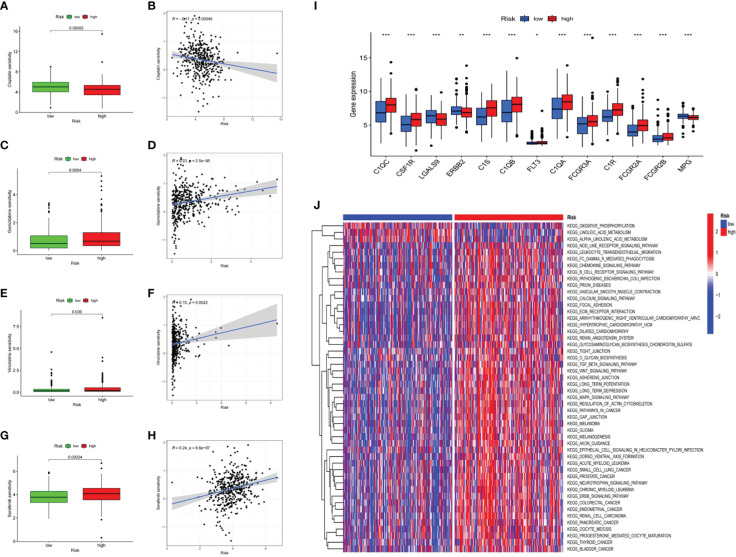
Prognostic risk score signature in the role of prediction in sensitiveness of chemotherapy and targeted therapy The response differences between low- and high-risk score groups to Cisplatin **(A)**, Gemcitabine **(C)**, Vincristine **(E)**, Sorafenib **(G)**. The association between risk scores of patients and drug sensitivity of Cisplatin **(B)**, Gemcitabine **(D)**, Vincristine **(F)**, Sorafenib **(H)**. **(I)** The boxplot of potential target genes in low- and hgh-risk score groups. *p < 0.05; **p < 0.01; ***p < 0.001. **(J)** The heatmap of GSVA enrichment between low- and high-risk score groups.

The gene sets represented in “c2.cp.kegg.v7.4” retrieved from MSigDB were used to conduct GSVA enrichment analysis. The results indicated different biological behaviors in the low-risk and high-risk groups, and several crucial metabolic pathways, including linoleic acid metabolism, alpha-linoleic acid metabolism and the citrate cycle, were enriched in the low-risk group (**Figure 6J**). Moreover, some pathways associated with immune biological processes, including the T-cell receptor, B-cell receptor and chemokine signaling pathways (**Figure 6J**), were enriched in the high-risk group, consistent with the analysis of immune infiltration. In addition to immunotherapy, chemotherapy and targeted therapy are also important treatments for bladder cancer patients and can significantly improve patient prognosis. Therefore, the relationship between risk score and resistance to therapy was explored. Sensitivity to multiple drugs was calculated using the “oncoPredict” R package to predict the therapeutic response. As shown in **Figure 6**, patients with high risk scores were relatively insensitive to the chemotherapeutic agents gemcitabine and vincristine and to the targeted therapy agent sorafenib, all of which are widely used in the treatment of patients with advanced BLCA. To screen for potential therapeutic agents for BLCA based on the CRG prognostic signature, we further analyzed the expression of drug target genes in the low-risk and high-risk groups. The results demonstrated that the response rates to cetuximab (C1R, C1S, C1QA, C1QB, C1QC, FCGR2A, FCGR2B and FCGR3A), cisplatin (MPG), trastuzumab (ERBB2) and sunitinib (CSF1R) were significantly higher in the high-risk group than in the low-risk group (**Figure 6I**).

### 2.7 Validation of the expression of prognosis-related CRGs in bladder tissues

**Figure 7:**
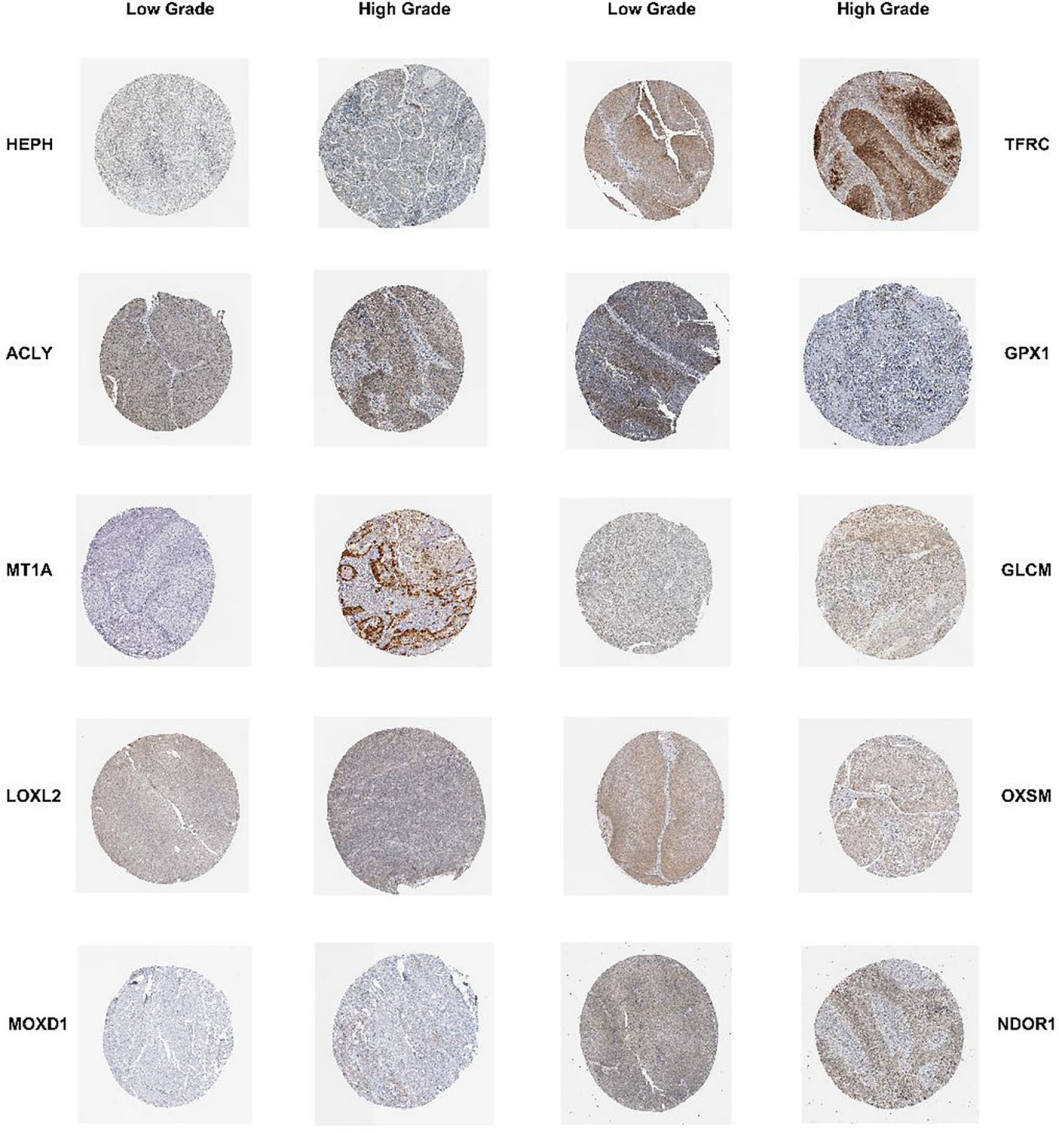
Validation of the 14 prognostic CRGs in protein level The immunohistochemistry staining results of HEPH, ACLY, MT1A, LOXL2, MOXD1, TFRC, GCLM, GPX1, OXSM, NDOR1 between low-grade and high-grade BLCA tissues from the HPA database.

To verify the differential expression and the prognostic value of the 14 CRGs, we next compared the protein expression of the above 14 CRGs in the high-risk and low-risk groups as reported in the HPA database. The results of immunohistochemical staining indicated that, compared to the samples in the low-risk group, the protein expression of HEPH, ACLY, MT1A, LOXL2, MOXD1, TFRC and GCLM was obviously elevated in the samples in the high-risk group. Furthermore, the protein expression of GPX1, NDOR1, and OXSM was elevated in the tissue samples from the low-risk group. The HPA database did not report the protein expression of S100A5, SNCB, SNAI5, or STEAP4 (**Figure 7**).

### 2.8 Identification and exploration of MOXD1 in depicting the tumor immune landscape

**Figure 8:**
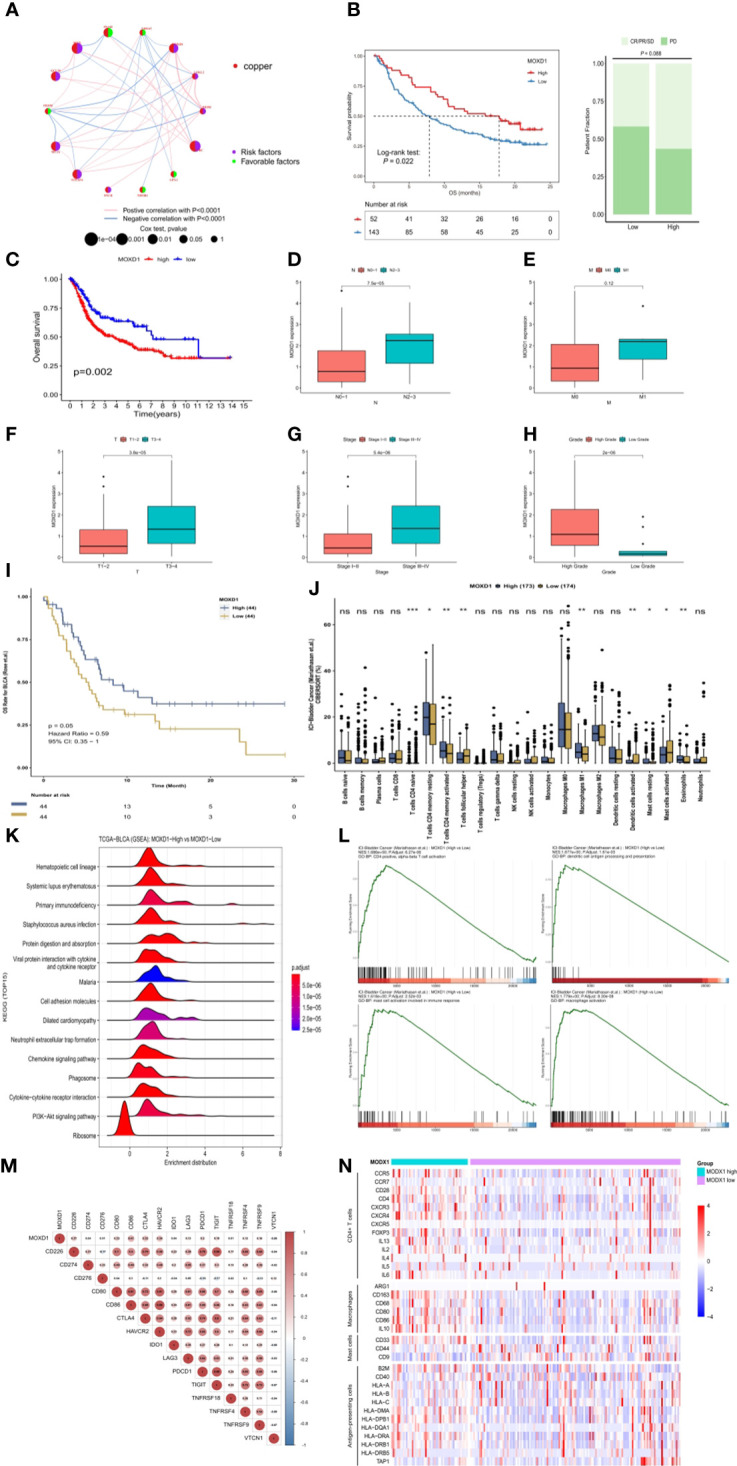
Identification and exploration of the MOXD1 in depicting tumour immune landscape **(A)** The network plot of interactive relationships, regulation bonds and their correlation significance between the 14 CRGs in BLCA patients. **(B)** The comparison of overall survival (OS) and clinical response between low- and high-expression of MOXD1 in the IMvigor210 cohort. **(C–H)** The comparison of overall survival and clinical characteristics in the TCGA-BLCA cohort. **(I)** Kaplan-Meier analysis with the log-rank test of high and low expression group of MOXD1. **(J)** Analysis of immune cell infiltration in bladder cancer samples with high- and low- expression in Mariathasan cohort. ns p>0.05; *p < 0.05; **p < 0.01; ***p < 0.001. **(K, L)** Gene Set Enrichment Analysis (GSEA) of the high and low expression level of MOXD1 groups. **(M, N)** The correlation analysis between MOXD1 and immune checkpoint inhibitor genes, and the differences of expression levels of surface markers (CD4 T cells, mast cells, macrophages, antigen presenting cells) between the high and low MOXD1 expression group in the IMvigor210 cohort.

First, the interactive relationships, regulation bonds and the significance of the correlations in the 14 CRGs in BLCA patients were depicted in a network plot (**Figure 8A**). To further identify genes that are closely associated with immune characteristics for subsequent study, we analyzed the survival and clinical outcomes associated with expression of the above 14 genes based on the expression profiles found in samples from bladder cancer patients in the IMvigor210 dataset who had been treated with immunotherapy. The results showed that only one gene, MOXD1, was significantly associated with differential survival in the IMvigor210 cohort, and its expression was correlated with the patients’ clinical response (**Figure 8B**). MOXD1 was therefore used to explore the association between gene expression and clinical outcome (**Figure 8C**) as well as clinical characteristics (**Figures 8D-H**) in the TCGA-BLCA cohort. In addition, to further explore the predictive value of MOXD1 regarding the response to immunotherapy in bladder cancer patients, we utilized the Comprehensive Analysis on Multi-Omics of Immunotherapy in Pan-cancer (CAMOIP) database to analyze the immune characteristics of the patients in the groups with high and low expression of MOXD1. The results indicated that the MOXD1 expression level of samples in the Rose cohort increased as the risk of death from bladder tumors decreased (**Figure 8I**), consistent with the results obtained using the IMvigor210 cohort. An analysis of immune cell infiltration in patients with bladder cancer in the Mariathasan cohort is shown in **Figure 8J**. The group with high expression of MOXD1 showed remarkably poor cell population infiltration by activated dendritic cells and activated mast cells. Although there was no significant difference in CD8 T cells between the two groups, CD4 T cells were activated in all populations of samples with high MOXD1 expression (**Figure 8J**). A GSEA comparison of the groups with high and low expression of MOXD1 was also conducted; the resulting top 15 significant pathways are depicted in a ridge plot (**Figure 8K**). The significant pathways include the primary immunodeficiency and chemokine signaling pathways. The GSEA enrichment analysis also showed that the group with high expression of MOXD1 had greater enrichment of cell-related pathways that differed significantly with respect to immune cell infiltration (**Figure 8L**). Correlations between the expression of MOXD1 and the expression of immune checkpoint inhibitor genes (**Figure 8M**) and differences in the expression levels of surface markers for CD4 T cells, mast cells, macrophages, and antigen-presenting cells between the high and low MOXD1 expression groups were analyzed and validated in the IMvigor210 cohort (**Figure 8N**). It was confirmed that the group with high expression of MOXD1 had a promising immune response to immunotherapy.

### 2.9 High expression of MOXD1 in fibroblasts promoted an active tumor immune microenvironment according to scRNA analysis

**Figure 9:**
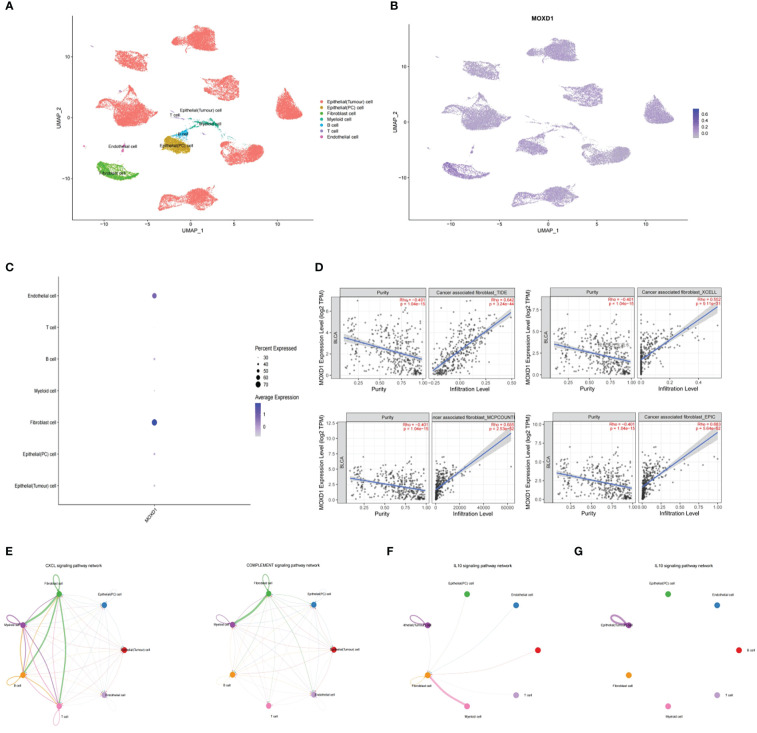
Stromal MOXD1 interacts with immune cells in bladder cancer **(A)** UMAP plot displaying the composition of 7 main cell clusters derived from bladder cancer samples. **(B)** UMAP plot displaying the expression level of MOXD1 in the whole cell clusters. **(C)** Bubble diagram showing the expression distribution of MOXD1 in the whole cell clusters. **(D)** Graph of correlation between MOXD1 and fibroblast fractions in bladder cancer from TIMER database. **(E)** Circos plots showing putative ligand-receptor interactions between fibroblasts and other cell clusters. **(F, G)** Circos plots showing individual ligand-receptor pairs between fibroblasts and immune cell clusters of tumour microenvironment with high-MOXD1 **(F)** and low-MOXD1.

We then attempted to explore the localization of MOXD1 in the tumor microenvironment and its specific impact on the tumor immune microenvironment (TIM) by analyzing the single-cell analysis expression profiles of bladder cancer in the GSE135337 dataset. Seven primary tumor tissue samples and one normal tissue sample were clustered and annotated into seven clusters, including epithelial (precancerous) cells, epithelial (tumor) cells, endothelial cells, T cells, B cells, myeloid cells, and fibroblasts (**Figure 9A**). After dimensionality reduction, 14 candidate copper-related genes were found to be predominantly highly expressed in fibroblasts and endothelial cells; moreover, MOXD1 was significantly highly expressed in fibroblasts (**Figures 9B, C**). To further verify the localization of MOXD1 in fibroblasts in bladder cancer tissue, we explored the correlation of MOXD1 expression with fibroblasts in the TIMER 2.0 database. The results demonstrated that under the TIDE, XCELL, EPIC, and MCPCOUNTER algorithms, MOXD1 expression was positively correlated with tumor infiltration by fibroblasts (**Figure 9D**). Furthermore, we explored pathways associated with ligand−receptor signaling among various cell populations by cell−cell communication analysis. The results demonstrated that the CXCL signaling pathway and the complement signaling pathway in fibroblasts were closely related to T cells, B cells, and myeloid cells (**Figure 9E**), suggesting that fibroblasts are a crucial cellular component in the progression of bladder cancer. Through analysis of the signaling pathway, we found increased ligand−receptor signaling between fibroblasts and T and B cells through the CXCL12-CXCR4 and CXCL2-CXCR2 axis in bladder cancer tissues as well as enhanced signaling from fibroblasts to myeloid cells, including signaling involving C3-CR2, C3-(ITGAX+ITGB2), C3-(ITGAM+ITGB2), and C3-C3AR1. The interactions indicated that fibroblasts might recruit T cells and B cells and regulate the proliferation and activation of macrophages (**Figure S3**). In addition, we found that interactions between fibroblasts and immune cells through the IL-10 signaling pathway were significantly increased in the tumor microenvironment in patients with high MOXD1 expression (**Figure 9F**) compared with patients with low MOXD1 expression (**Figure 9G**).

### 2.10 Knockdown of MOXD1 in fibroblasts significantly inhibited the proliferation and migration of BLCA cells

**Figure 10:**
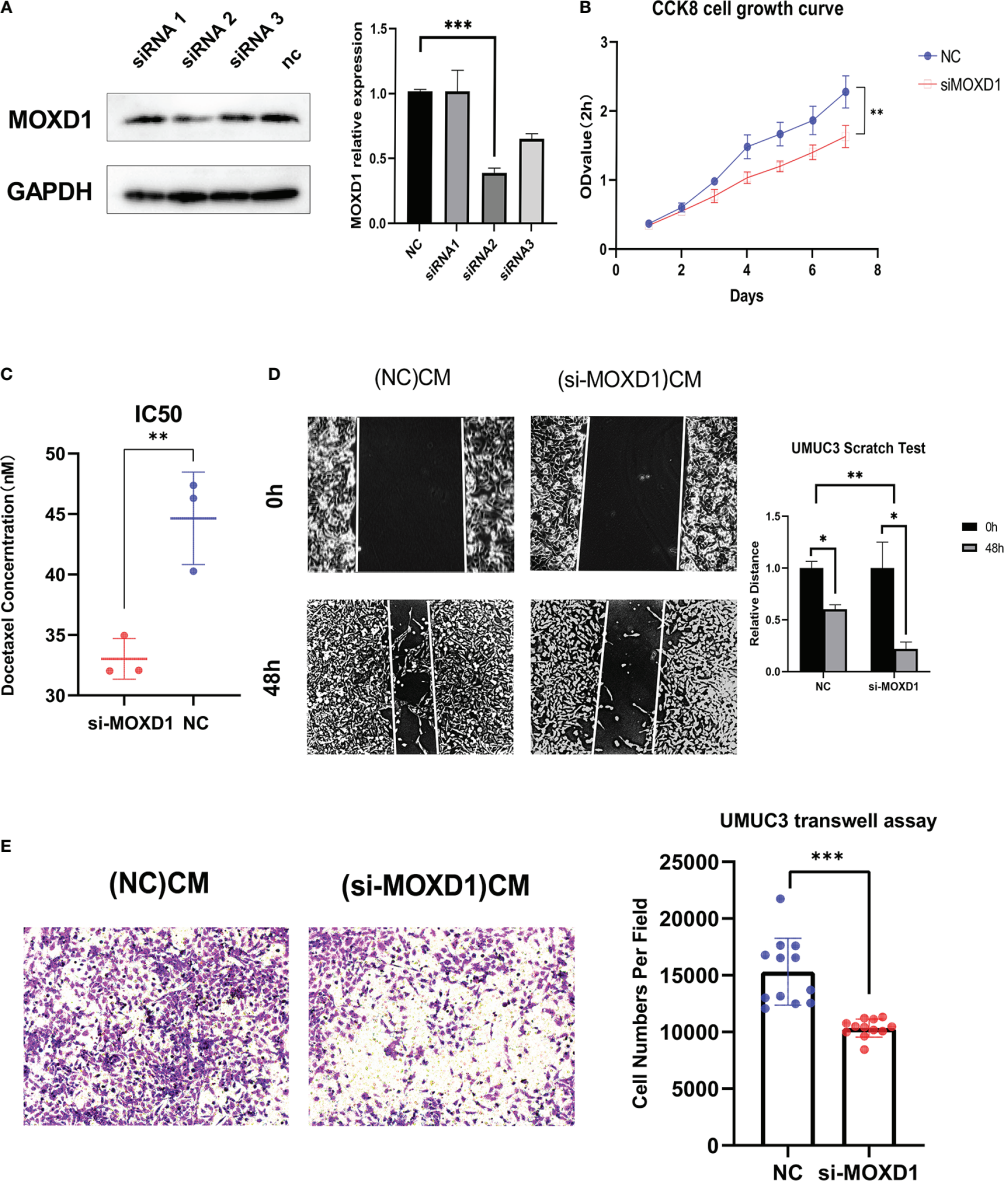
*In vitro* experiments **(A)** Relative protein level of MOXD1 in fibroblasts following siRNA knockdown. GAPDH served as loading control. Statistical graphs are presented as bar plot. **(B)** The CCK8 cell growth experiment was used to analyze the influence of stromal MOXD1 on the proliferation ability of UMUC3 cells. The results are presented as the mean optical density (OD) at 450 nm for triplicate wells two hours after the incubation. The results are presented as the mean ± SD of three independent experiments (*p < 0.05, **p < 0.01, ***p < 0.001). **(C)** The Docetaxel resistance of stromal MOXD1 for UMUC3 cells was then measured by the IC50 assay, and the result showed that si-MOXD1 fibroblasts decreased the IC50 value of UMUC3, comparing to negative control group (Figure 10C). **(D)** The scratch wound assay determined that knock-down of MOXD1 in fibroblasts attenuated the migration ability of UMUC3 cells. The quantifications of cell migration were presented by the histogram. **(E)** Transwell assay indicated that knock-down of MOXD1 in fibroblasts weakened UMUC3 cells invasion. The quantifications of cell migration were presented by the column chart.

The role of MOXD1 was validated in *in vitro* experiments. MOXD1 expression in fibroblasts was knocked down using siRNA-MOXD1; the results are shown in **Figure 10A**. siRNA 2 yielded the best knockdown efficiency, and it was selected for use in subsequent experiments. CCK8 cell growth experiments showed that conditioned medium from MOXD1 knockdown fibroblasts inhibited the proliferation of BLCA cells (**Figures 10B**, **S4A**). The docetaxel resistance of UMUC3 cells was then measured using an IC50 assay; the results showed that exposure to conditioned medium from si-MOXD1 fibroblasts decreased the IC50 of UMUC3 cells for docetaxel compared that of to negative control UMUC3 cells (**Figure 10C**). A scratch wound assay was used to determine the effect of MOXD1 on the migratory ability of BLCA cells. MOXD1 expression in fibroblasts increased the migratory ability of UMUC3 and 5637 cells (**Figures 10D**, **S4B**). The migratory ability of UMUC3 cells was also determined using a Transwell assay, and the results were consistent with those obtained using the scratch wound assay (**Figure 10E**).

## 3 Discussion

Because copper participates in many cancer-associated biological processes, including mitochondrial respiration, antioxidant defense, mitogenic signaling and autophagy, abnormalities in copper metabolism are crucial in tumorigenesis, cancer progression and cancer therapy (16). Serum copper and zinc levels are closely related to the expression of HIF1-α and VEGF in bladder cancer tissue, indicating that copper plays an important role in angiogenesis. Logistic regression analysis suggests that increased plasma copper is a risk factor for the development of bladder cancer (17). It has been reported that copper transporter receptor 1 (CTR1) influences sensitivity to platinum-based neoadjuvant chemotherapy for bladder muscle-invasive bladder cancer (18). However, almost all studies conducted to date have been limited to individual genes, and few comprehensive studies have focused on the relationship between copper and bladder cancer. Our study provides a robust risk prediction signature for bladder cancer and may help investigators understand the role of copper metabolism in bladder cancer progression and lead to more in-depth studies.

We constructed a prognostic risk signature for predicting the OS of bladder cancer patients in the TCGA cohort through Cox regression analysis and LASSO Cox regression analysis and validated it in the GEO database; thus, this signature can be used to screen patients for low survival. Our signature is also an independent prognostic factor for bladder cancer. A nomogram that integrates several specific clinical features was constructed to enhance the predictive power of the model. In addition, using gene cluster analysis, we further evaluated the value of the genes identified using the prognostic model in predicting prognosis and risk in bladder cancer patients and confirmed the predictive advantage of establishing a prognostic model based on copper-related genes. In addition, using unsupervised cluster analysis, we confirmed that the 14 genes that constitute the prognostic model effectively distinguish bladder cancer patients with different prognostic outcomes.

Among the 14 identified candidate genes, HEPH, TFRC, and LOXL2 were the genes with the most frequent mutations. HEPH, a multicopper ferroxidase, is a membrane-bound homolog of ceruloplasmin; it contains copper-binding sites that are involved in the transport of iron from enterocytes. Low expression of HEPH results in dysregulation of iron homeostasis and is associated with the occurrence of colorectal carcinoma (18, 19). Additionally, aberrant expression of HEPH has been observed in BT-474 and T-47D breast cancer cells (19). TFRC, a cofactor for oxygen-carrying proteins, is crucial for cell proliferation, and a study of TFRC in brain tissue has shown that it regulates the transformation of ferrous iron to the ferric form, a process that is related to copper and zinc SOD levels (20). Aberrant iron ions may then result in the production of ROS, iron deposition, abnormal lipid peroxidation, and finally abnormal cell growth, apoptosis, and other biological events. In glutathione-S-transferase placental form-positive liver neoplastic lesions, the expression of TFRC is upregulated, and its increased expression may be a hallmark of enhanced ROS production (21). LOXL2, a copper-containing enzyme, is closely related to reduced survival time and poor prognosis since it promotes the proliferation, migration, invasion, and metastasis of numerous types of cancers, including breast cancer, lung cancer, colon cancer, and liver cancer. Increased expression of LOXL2 also results in reduced chemosensitivity in triple-negative breast cancer. A possible mechanism through which this may occur is the creation by LOXL2 of a collagen scaffold that helps disseminate cancer cells; copper depletion decreases collagen cross-linking, as measured by LOXL2 levels, thus preventing breast carcinoma metastasis. In certain types of cancer, this process is more copper-dependent (22–24). Another significant candidate gene is the gene that encodes six-transmembrane epithelial antigen of the prostate 4 (STEAP4). STEAP4 is a transmembrane protein that acts as a metalloreductase during the transport of copper, and it prevents cell oxidative stress by importing copper to the cytosol. Overexpression of STEAP4 is thought to increase ROS, and this may increase gene mutation frequency and the progression of prostate cancer (25).

We also used clinicopathological characterization in this study. As we expected, the cases in the TCGA cohort with higher risk scores had worse outcomes, including more metastatic lesions, more regional lymph node involvement, higher-grade tumors, more advanced tumor stage, and larger primary lesions and involved areas. This indicates that our signature is powerful in predicting the extent of tumor progression. Our results also show that the tumor microenvironment in patients with lower risk scores is more likely to contain CD8 T cells and activated dendritic cells and less likely to contain M2 macrophages. CD8+ T cells play a crucial role in the regulation of immune functions such as immune surveillance of tumor cells (26), and dendritic cells are needed for generation of antitumor immunity (27). The macrophage immune mechanism involves activated M1 and M2 macrophages and angiogenic and immunosuppressive molecules that inhibit the immune response to tumor cells (28). To evaluate whether our signature shows promise in predicting patient response to ICIs, we calculated TIDE scores for the high-risk and low-risk groups. The TIDE score reflects tumor immune escape in the context of different cytotoxic T lymphocyte levels and has been widely used to predict disease outcome in patients who have been treated with ICIs (29, 30). Patients with higher risk scores generally have higher TIDE scores, indicating that they are more vulnerable to immune dysfunction and escape. A number of recently published articles focusing on pyroptosis in bladder cancer also noted that OS, clinical outcome, and response to immunotherapy in bladder cancer patients with high cuproptosis risk were significantly worse than those in the low-risk group. These results are consistent with our results and suggest that copper metabolism, and even pyroptosis, should be considered when devising personalized treatment for patients with bladder cancer (1, 31).

To our surprise, the results of our study revealed that chemotherapy and target drug sensitivity differed in the high- and low-risk groups. Patients with lower risk scores tend to be more sensitive to most chemotherapeutic and targeted agents, such as gemcitabine, vincristine, and sorafenib. In contrast, patients in the high-risk group were more sensitive to cisplatin. Cisplatin can be used as a first-line agent in the treatment of bladder cancer, and its use considerably reduces the risk of bladder cancer-induced death (32). Gemcitabine, a commonly used intravesical chemotherapeutic drug, has significant efficacy and value in reducing the recurrence of non-muscle-invasive bladder cancer (33). Sorafenib is a multikinase inhibitor that inhibits tumor growth mainly through its anti-angiogenic effect, and a previous study showed that it has an inhibitory effect on the proliferation of human bladder cancer cell lines (34, 35). “OncoPredict” is a widely accepted R package that is used to predict drug responses based on gene expression levels. The drug sensitivities of the patients in our study were consistent with the patients’ risk scores after analysis of the 17 gene expression levels using “oncoPredict”, suggesting that the signature may help predict drug responses in the clinic.

We identified MOXD1 among 14 candidate genes analyzed by network construction and external validation. MOXD1 was the only candidate gene found to be significantly associated with overall survival in the IMvigor210 cohort. A monooxygenase, MOXD1 is primarily located in the endoplasmic reticulum, where it binds copper ions. In the TCGA cohort, high MOXD1 expression was associated with poorer overall survival; however, validation in immunotherapy cohorts showed that patients with high MOXD1 expression had longer overall survival. The results suggested that high expression of MOXD1 in the patients in the TCGA cohort who were treated with methods other than immunotherapy, such as surgery alone or chemotherapy, is indicative of poor response and survival and that these individuals may be better candidates for treatment with immunotherapeutic agents such as immune checkpoint inhibitors. Based on further exploration through single-cell analysis, we also found that MOXD1 is mainly overexpressed in fibroblasts, suggesting that fibroblasts may play an important role in shaping the immune microenvironment in bladder cancer by changing the transport and activity of copper ions.

This study has some shortcomings. First, the data used in our analysis were retrieved from public databases and are therefore likely to have selection bias that may influence the accuracy of the analysis to some extent. In addition, the effect of MOXD1 expression in stromal cells on immune cells is unknown. Therefore, to increase the clinical significance of our findings, in the future we will conduct an in-depth study of the influence of copper-related gene expression in stromal cells on immune cells and combine it with a study of clinical phenomena.

## 4 Materials and methods

### 4.1 Data preparation and processing

The original transcriptome sequencing (RNA-seq) data profiles of BLCA and normal bladder tissue samples were obtained from TCGA database (https://www.cancer.gov/about-nci/organization/ccg/research/structural-genomics/tcga) in the format of fragments per kilobase of transcript per million mapped reads (FPKM). The 10 copper-related gene sets with the largest number of genes were downloaded from MigSDB (https://www.gsea-msigdb.org/gsea/msigdb) and were incorporated into CRGs gene sets. The clinical information of 405 BLCA samples, including age, sex, pathological stage, grade, AJCC TNM stage, and survival outcome, was also downloaded from the TCGA database. The microarray data profiles and clinical information of GEO: GSE13507 with complete clinical outcomes were downloaded from the GEO database (https://www.ncbi.nlm.nih.gov/geo/). Annotated by platform GPL6102, the Entrez Gene IDs were correspondingly transformed into gene symbols. If multiple probes were targeted to identical Entrez gene IDs, the average value was adopted. The copper-related gene sets with the top ten largest gene numbers were retrieved from MigDSB database. After merging these gene sets, duplicate genes were deleted to develop a target gene set containing 180 genes (**Table S1**), and the expression levels of these genes were extracted from the TCGA training set and GEO validation set.

### 4.2 Difference analysis of the copper-related genes in the normal and tumour tissue samples

The “limma” package in R was used to determine the copper-related DEGs in the normal bladder and BLCA tissue samples. Statistically significant genes were considered by FDR < 0.05. The conversion of the gene symbols into Entrez Gene IDs was conducted by “org.Hs.e.g.db” package in R. The “pheatmap” package in R was further used to depict the volcano plot and heatmap of DEGs.

### 4.3 Construction and validation of a copper-related prognostic risk score signature

TCGA cohort samples were included in the training set, and the test set was constructed by GEO: GSE13507 cohort samples. First, the differentially expressed copper-related gene expression data were combined with the corresponding prognostic information based on the ID of the samples. Through univariate Cox regression analysis in the training set, the genes related to prognosis with a p value <0.05 were screened from copper-related DEGs. The mutation and comutation in the prognosis-related genes of the TCGA cohort were analysed by the “maftools” R package. Furthermore, LASSO Cox regression analysis was utilized to develop a prognostic risk signature including the prognosis-related genes for predicting the OS of BLCA samples by the “glmnet” R package. Tenfold cross verifications were performed to determine the penalty parameter (λ) of the signature. The formula below was used to calculate the risk score for the corresponding sample.

Risk Score = (Gene 1 expression × coefficient) + (Gene 2 expression × coefficient) + … + (Gene n expression × coefficient)

In the LASSO Cox regression analysis, “coefficient” refers to the nonzero regression coefficients, in addition, “Gene n expression” represents the prognosis-related gene expression values. According to the median risk score, the samples were classified into low- and high-risk groups. Kaplan-Meier analysis with the log-rank test was used to compare the difference in OS and PFS between the low- and high-risk score groups in the training set, which was further validated by the OS of the low- and high-risk score groups in the test set. The predictive accuracy of the prediction signature was proven by plotting the time-dependent ROC curve with the “survivalROC” R package. In the samples of the TCGA cohort, the risk score and clinical information were combined by the sample ID. The limma package in R was used to analyse the correlation between the risk score and clinical characteristics, including sex, age, grade, tumor stage, and AJCC TNM stage. Significant differences were confirmed with a p value < 0.05.

### 4.4 The analysis of principal-component with or without prognostic risk score signature

The “limma” R package was comprehensively used to perform PCA on expression profiles in the TCGA cohort with all differentially expressed copper-related genes and genes obtained from the signature to demonstrate the significant discrimination of all samples by the prognostic risk score signature. Conculsively, using the ggplot2 R package, the PCA results presented a prominent two-dimensional distinction with from the first two principal components.

### 4.5 Consensus clustering analysis of 14 prognosis related CRGs

Based on the expression profiles of 14 CRGs, the “ConsensusCluster Plus 1.60.0” package in R was used to estimate the unsupervised classes of TCGA-BLCA dataset using the consensus clustering method, and two clusters A and B were obtained respectively (36). Kaplan-Meier analysis with the log-rank test was used to compare the difference in OS between the different clusters. The “pheatmap” package in R was further used to depict the heatmap of gene based on different clusters. The “ggalluvial” package in R was performed to depict the alluvial diagram of clinical outcomes, risk scores, CRGclusters and geneclusters.

### 4.6 GSVA

GSVA, a nonparametric and unsupervised method, was performed by the “gsva” R package on the sample expression matrix to compare the variations in functional pathways between the low- and high-risk groups (37). The reference gene lists, “c2.cp.kegg.v7.4.symbols” gene sets, were obtained from the molecular signatures database (https://www.gsea-msigdb.org/gsea/msigdb). Enrichment pathways with significant differences are indicated by FDR <0.05.

### 4.7 Features comparison of the low- and high-risk score groups

The sensitivity of chemotherapy drugs and target drugs in each sample were predicted using the oncoPredict R package (38). The “GSEABase” and GSVA R packages were used for performing ssGSEA to depict the immune-infiltrated status of samples in the training set, which incorporated immune cell groups and corresponding activities in the TME, such as activated dendritic cells, M2 macrophage, parainflammation (Table S3) (39, 40). The definition of C1-C4 immune subtype was deriveded from previous study (30). The distinction of immune-infiltrated status in the groups with low- and high-risk scores was compared according to the enrichment scores calculated by the ssGSEA algorithm. Conclusively, the TIDE (http://tide.dfci.harvard.edu/) algorithm was used to predict and compare the different responses to ICIs in low- and high-risk groups. A response with a significant difference was indicated by a p value < 0.05. The “ESTIMATE” package in R was used to calculate the ImmuneScore, StromalScore, and EstimateScore. Cancer stemness was computed by RNAss.

### 4.8 Validation of MOXD1 gene in immune checkpoint inhibitor treatment cohorts

Expression profile of 195 ICI-treated bladder urothelial carcinoma samples in IMvigor cohort was downloaded from (http://research-pub.gene.com/IMvigor210CoreBiologies/.RNA-seq) along with the relevant clinical data. Kaplan-Meier analysis with the log-rank test was used to compare the difference in OS between the low- and high-risk score groups with a best cutoff value in the IMvigor cohort. Using the CAMOIP online database (version: 1.1; http://www.camoip.net/) (41), groups with different expression level of MOXD1 were analysed in Rose cohort and Mariathasan cohort (42, 43).

### 4.9 Construction of a nomogram for overall survival prediction

The “rms” R package was used to develop a nomogram incorporating by age, sex, tumour stage, grade, and a prognostic risk score signature for predicting OS in the TCGA cohort. The accuracy of the nomogram was represented by a time-dependent calibration curve. In addition, multivariate Cox regression analysis was performed to verify whether the prognostic risk score signature can be independently used as a predictor of OS in bladder cancer. Then, the AUC was calculated by an online ROC curve to represent the prognostic value of the nomogram.

### 4.10 Validation of protein expressions of 14 prognostic CRGs

The Human Protein Atlas (HPA) (https://www.proteinatlas.org/) database was used to validate the protein expression of 14 prognostic CRGs between low-grade and high-grade samples using immunohistochemistry (IHC).

### 4.11 Single cell RNA sequencing analysis

The scRNA-seq dataset (GSE135337) of bladder cancer was obtained from GEO database. The “DropletUtils” package (v 3.13) in R was performed to conduct quality control. The data normalization was performed using the NormalizeData function of the “Seurat” package. Then, cell populations were clustered using the FindNeighbors and FindClusters function of the “Seurat” package. A dimension reduction method-Uniform Manifold Approximation and Projection (UMAP), was performed to depict cell clusters. We analyzed intercellular communication networks from scRNA-seq data with “CellChat”, a package in R (44). TIMER 2.0 database (timer.cistrome.org) was used to analyzed the correlation of fibroblasts and MOXD1.

### 4.12 Cell culture

Human urothelial cell SVHUC-1 and bladder cancer cells UMUC3, 5637 were obtained from American Type Culture Collection Cell Biology Collection (ATCC, Manassas, VA, USA) and maintained in Department of Urology, Shanghai General Hospital (Shanghai, China). Cells were cultured in DMEM medium supplemented with 10% fetal bovine serum (FBS) at 37°C in a humidified incubator containing 5% CO2.

### 4.13 RNA extraction and real-time quantitative PCR

Total RNA was extracted from SVHUC-1 (normal urothelial cell line) and UMUC3 (bladder cancer cell line). RT-qPCR experiments were performed as previously described. The mRNA expression level of the 14 CRGs was calculated by the 2–ΔΔCt method and the results were plotted by using ACTB as the reference gene. The primers used in this study were shown in **Supplementary Table S4**.

### 4.14. Transfection, RNA interference and Western blot

SiRNA against human MOXD1 and control siRNA was purchased from GENE (Genechem, Shanghai, China). 2× 10^6^ fibroblast cells were plated in 6 wells dishes and infected with 50nM of siRNA using Lipofectamine™ 3000 (Invitrogen, Carlsbad, CA, USA) according to the manufacturer’s instructions. 36h after transfection, cells were lysed in RIPA for Western blot. Total cellular proteins were lysed by RIPA buffer containing protease inhibitor. Protein concentration were estimated by the BCA (Thermo Scientific), 40μg were loaded per lane on 10% SDS-PAGE and transferred onto polyvinylidene fluoride (PVDF) membranes (Millipore, USA). After blocked with 5% fat-free milk, the membranes were incubated with anti-MOXD1 antibody (1:1000, Abcam, USA) or anti-GAPDH antibody (1:1000, Abcam, USA) at 4 °C overnight. The membranes were then incubated with peroxidase (HRP)-conjugated secondary antibody (1:1000, Cell Signaling Technology, USA). Signals were visualized with Immobilon™ western chemiluminescent HRP substrate (Millipore) and analyzed by Image Lab Software.

### 4.15 Isolation and culture of fibroblasts

The fibroblasts were isolated from a human bladder biopsy. Briefly, after incubation in 500 μg/mL thermolysin at 4 °C overnight, the stroma was separated from the urothelium, then the stroma was treated by 0.125 U/mL collagenase H for 30 minutes at 37°C. The fibroblasts were enzymatically dissociated from the stroma and then, cultured in the DMEM (Gibco) supplemented with 10% FBS (Gibco) and 1% penicillin streptomycin (Gibco).

### 4.15 Conditioned medium preparation

First, 5 × 10^6^ bladder fibroblasts were plated on 10-cm dishes in regular growth media and allowed to adhere overnight. After the knockdown with siRNA, the supernatant was collected after culturing in fresh medium supplemented with 10% FBS for 48 h; then the supernatant was collected and centrifuged at 5000 g for 10 min, filtered with 0.22 μm filters and kept at −80°C until use.

### 4.16 CCK-8 assay and IC50 assay

Cell survival rates were assessed by CCK-8 assay (HY-K0301, MCE) following the manufacturer’s instructions. Briefly, approximately 2000-10000 cells were seeded into 96-well plates with 100μL medium, after 24h, a 10μL CCK-8 solution was added to each well. The absorbance at 450 nm of each well were measured in a microplate reader after the plates incubated for an additional 1h away from light. For IC50 assay, UMUC3 cells were seeded in to 96-well plates at a density of 2000-5000 cells per well for 24h, then different concentration of 0, 1, 10, 50, 100, 200 nM Docetaxel contained in 100 μL si-MOXD1 fibroblast conditioned medium or si-NC fibroblast conditioned medium were incubated for a further 48h, cell viability was tested by CCK-8 assay.

### 4.17 Transwell migration assay

Cell migration was determined by Transwell (Costar) migration assay. UMUC3 cells and 5637 cells were precultured in serum-free medium for 24 h. 1 × 10^4^ cells suspended in 100μl were seeded in serum-free medium in the upper chamber, and 900 μl si-MOXD1 fibroblast conditioned medium or si-NC fibroblast conditioned medium with 10%FBS was added to the lower chamber. After 24 h, the non-migrating cells on the upper chambers were carefully removed, and migrated cells underside of the filter were stained with 0.1% crystal violet and counted in six different fields.

### 4.18 Cell scratch test

UMUC3 and 5637 cells were seeded in 6-well plate, cultured with si-MOXD1 fibroblast conditioned medium or si-NC fibroblast conditioned medium for 48-72 hours, when the cells reached a confluent state, a single scratch was made using a sterile 200 μl tip. The floating cells were washed three times with PBS, and serum-free DMEM was added in the well. The image of the scratch was captured before and after incubation in a 37°C and 5% CO_2_ incubator.

### 4.19 Statistical analysis

Comparisons between two groups were calculated by the Wilcoxon rank sum test, and comparisons among three or more groups were performed by the K-W test (P < 0.05). Kaplan-Meier analysis was conducted in the low- and the high-risk score groups to assess the difference in prognosis. Moreover, the independent predictors of OS in bladder cancer were identified by multivariate Cox regression analysis. ROC curves were used to assess the accuracy of the prediction ability of the prognostic risk score signature and nomogram. R 4.0.5 for all statistical analyses.

## 5 Conclusions

In summary, this study depicted the landscape of crucial copper-related genes in BLCA. We identified two molecular subtypes of copper-related genes and constructed a copper-related genes signature. The prognosis of BLCA patients was predicted by the copper-related gene prognostic scoring system. There were significant differences in TIM and drug sensitivities between high and low score patients. In addition, we further screened the MOXD1 gene as a key node affecting the relationship between copper related genes and immune characteristics in tumor tissues of BLCA patients. The MOXD1 and copper-related gene prognostic scoring system could be helpful to understand the tumor characteristics of BLCA and develop personalized immunotherapy strategies.

## Data availability statement

The original contributions presented in the study are included in the article/supplementary material. Further inquiries can be directed to the corresponding authors.

## Author contributions

Conceptualization, WW, SH. Methodology, WW, SH, JL, JZ, and YZ. Validation, WW, SH, JZ, and YZ. Writing—original draft preparation, WW, SH. Writing—review and editing, WW, SH, JZ, and YZ. Supervision, JJ, BH. Funding acquisition, BH. All authors have read and agreed to the published version of the manuscript.

## Supplementary material

Supplementary Figure 1 The relationship between risk scores and survival status of samples in the training set and validation set **(A)** In the training set, the distribution of risk scores ranked from low to high; **(B)** the comparison of survival status between low- and high- risk score groups. **(C)** In the validation set, the distribution of risk scores ranked from low to high; **(D)** the comparison of survival status between low- and high- risk score groups.

Supplementary Figure 2 Correlation analysis of risk score and tumour characteristics **(A)** Correlation analysis of risk score and immune cell infiltration in TCGA-BLCA cohort. **(B)** Correlation analysis of risk score and 14 prognostic CRGs in TCGA-BLCA cohort. **(C)** Comparisons of immune landscape between low- and high- risk group from ICIs-treated patients based on TIDE algorithms.

Supplementary Figure 3 Cell-cell communication analysis of the tumour microenvironment. Interactions between cell clusters in CXCL signaling pathway and Complement signaling pathway.

Supplementary Figure 4 Cellular function experiments in 5637 cell line **(A)** The CCK8 cell growth experiment was used to analyze the influence of stromal MOXD1 on the proliferation ability of 5637 cells. The results are presented as the mean optical density (OD) at 450 nm for triplicate wells two hours after the incubation. The results are presented as the mean ± SD of three independent experiments (* p < 0.05, * * p < 0.01, * * * p < 0.001). **(B)** The scratch wound assay determined that knock-down of MOXD1 in fibroblasts attenuated the migration ability of 5637 cells. The quantifications of cell migration were presented by the histogram.
