# Supplementary figures and images for: Tumour microenvironment landscape and immunotherapy response in bladder cancer decoded by stromal MOXD1 based on copper-related genes signature
Source: Front Oncol. 2022 Dec 22;12:1081091. doi: 10.3389/fonc.2022.1081091 (PMC9815449; doi:10.3389/fonc.2022.1081091)

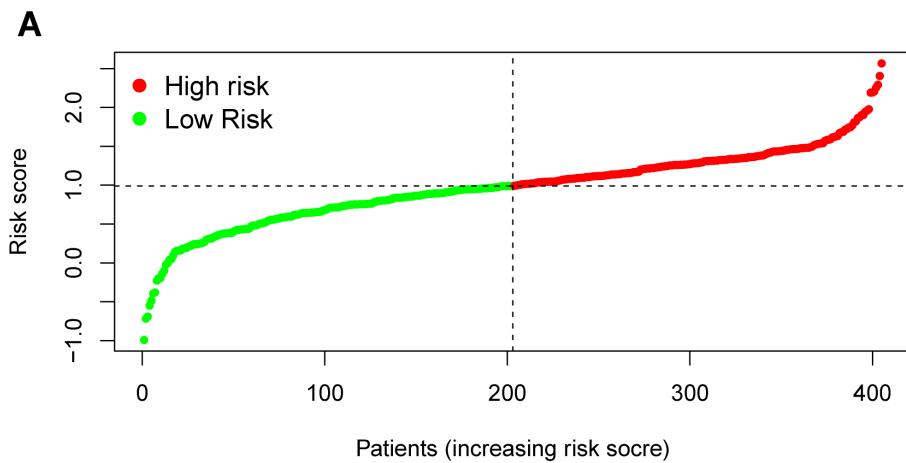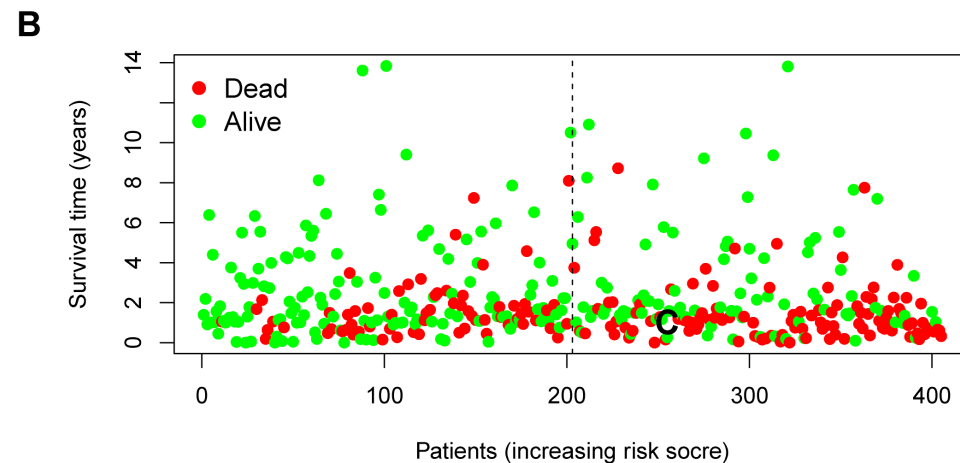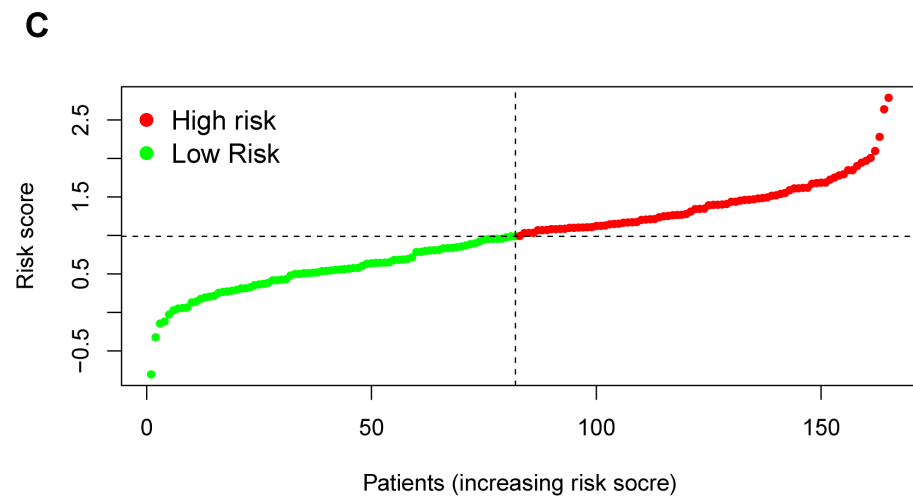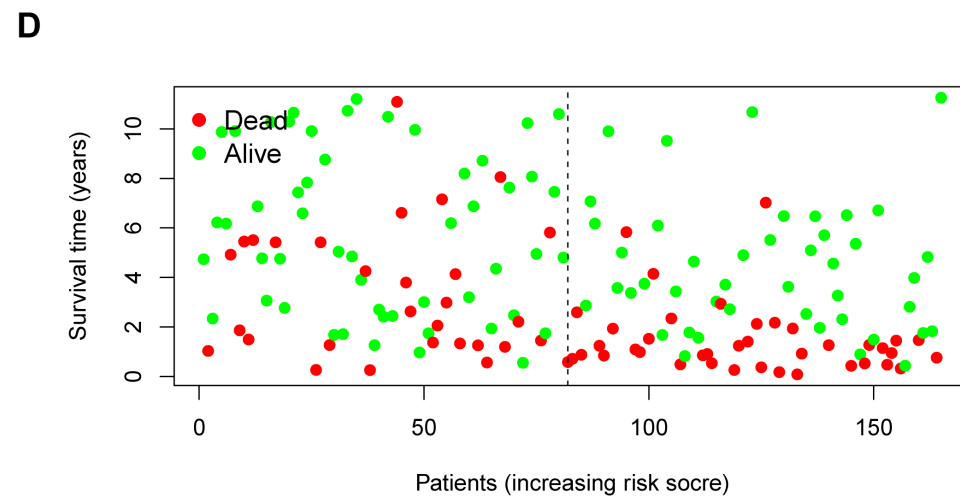

Supplement: Supplementary Figure 1 — The relationship between risk scores and survival status of samples in the training set and validation set (A) In the training set, the distribution of risk scores ranked from low to high; (B) the comparison of survival status between low- and high- risk score groups. (C) In the validation set, the distribution of risk scores ranked from low to high; (D) the comparison of survival status between low- and high- risk score groups. [file Image_1.pdf]

# A

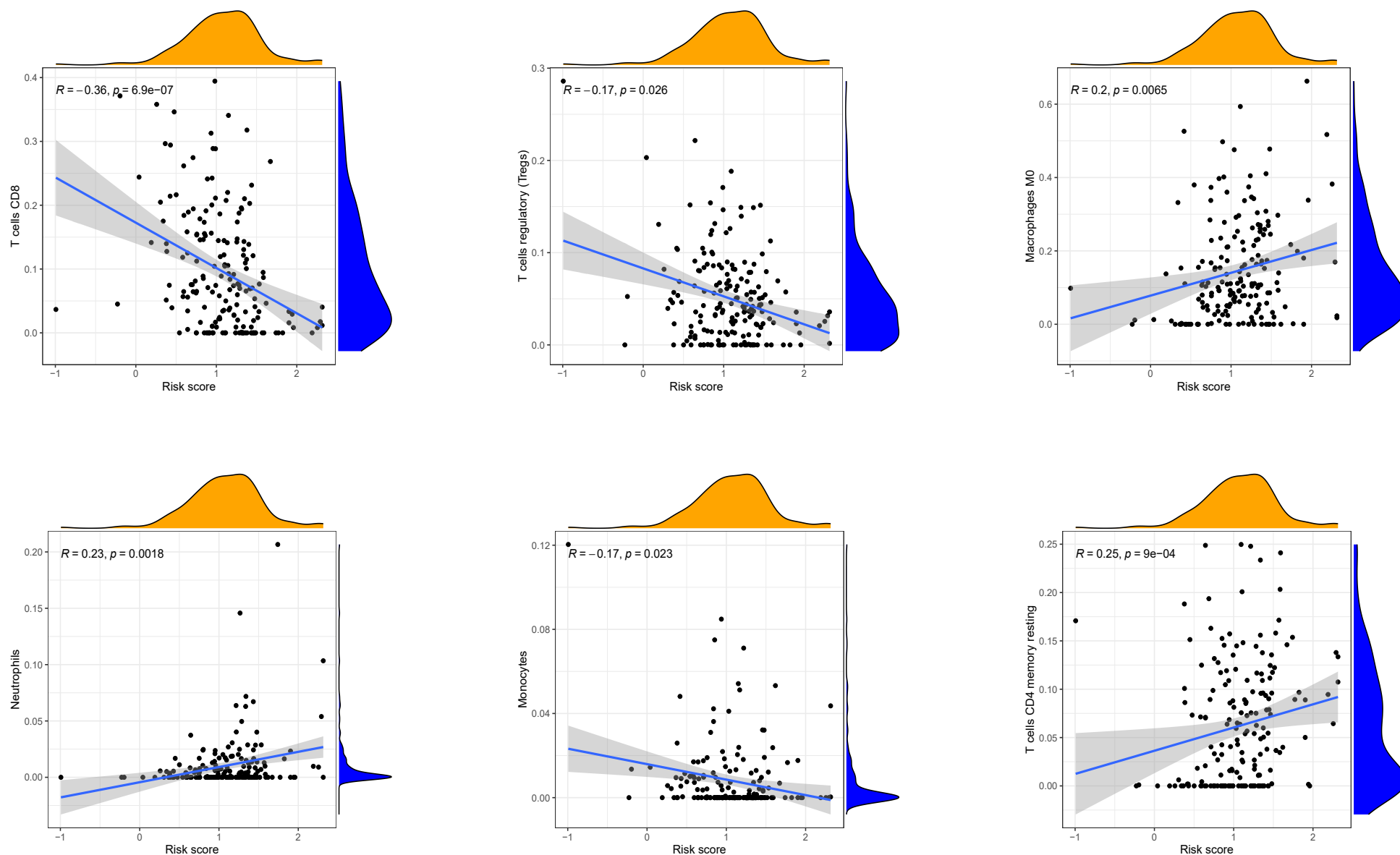

# B

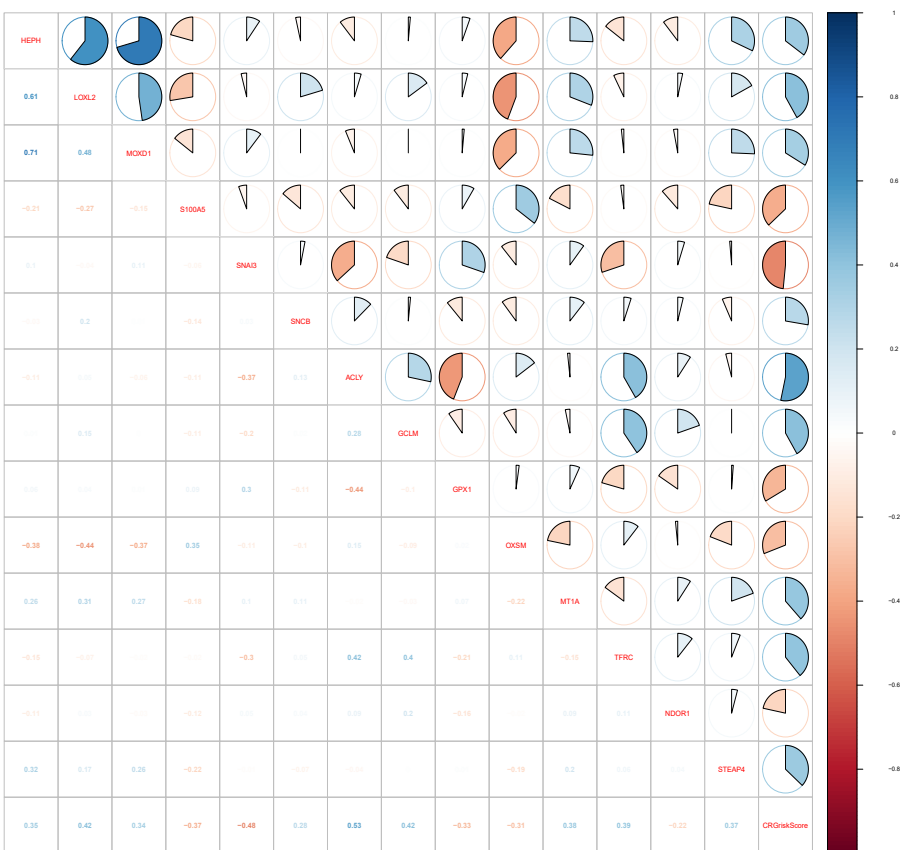

# C

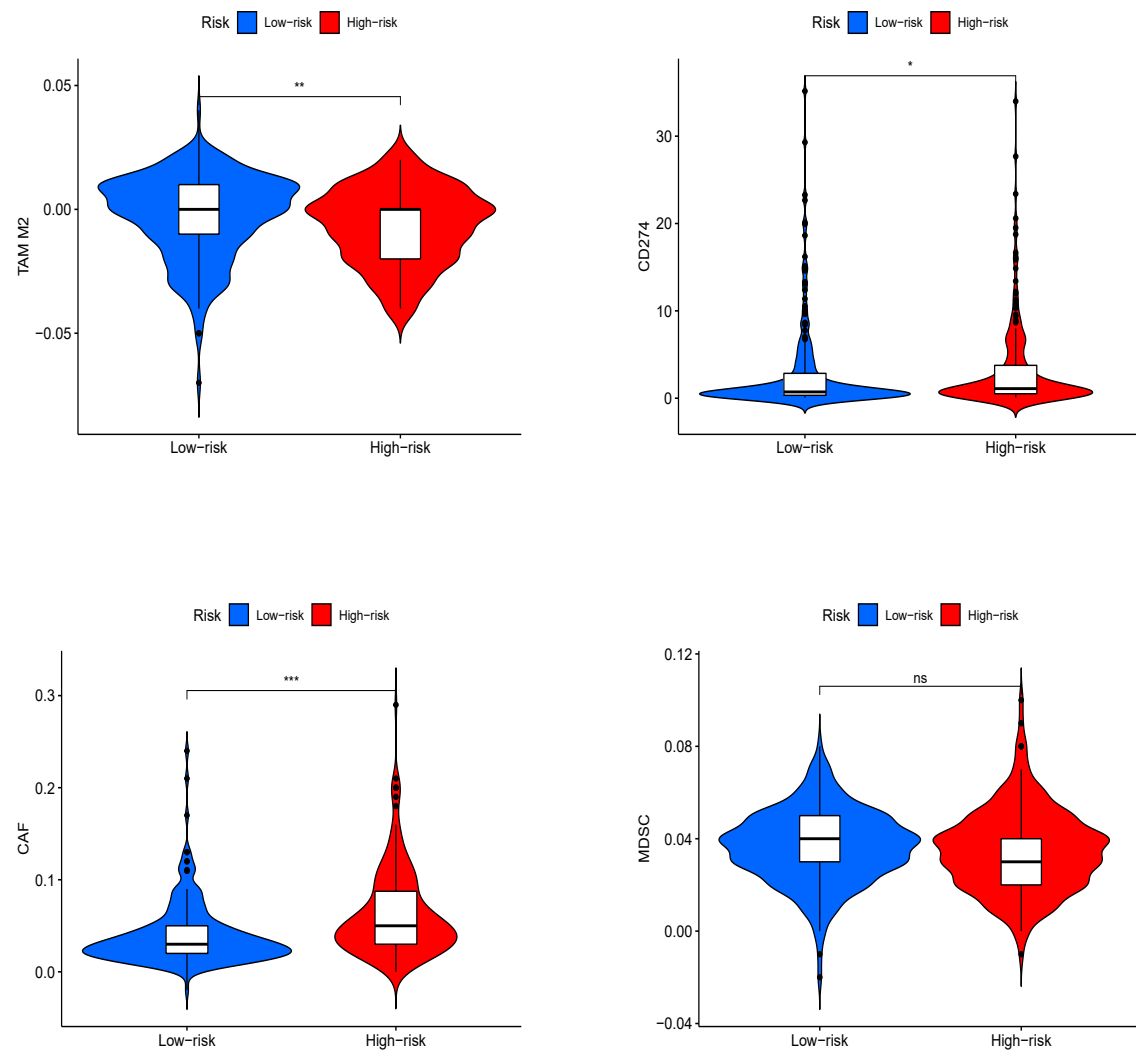

Supplement: Supplementary Figure 2 — Correlation analysis of risk score and tumour characteristics (A) Correlation analysis of risk score and immune cell infiltration in TCGA-BLCA cohort. (B) Correlation analysis of risk score and 14 prognostic CRGs in TCGA-BLCA cohort. (C) Comparisons of immune landscape between low- and high- risk group from ICIs-treated patients based on TIDE algorithms. [file Image_2.pdf]

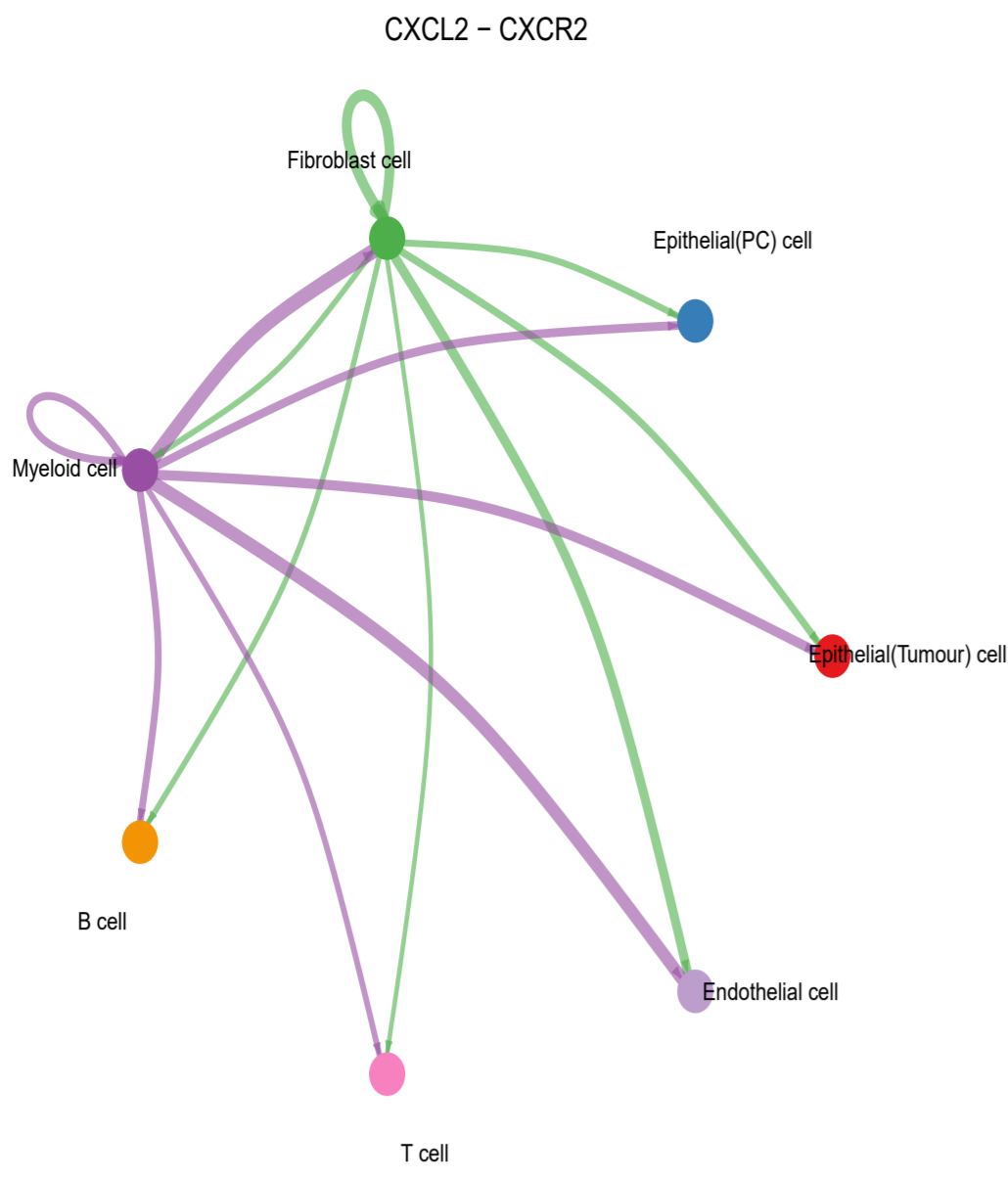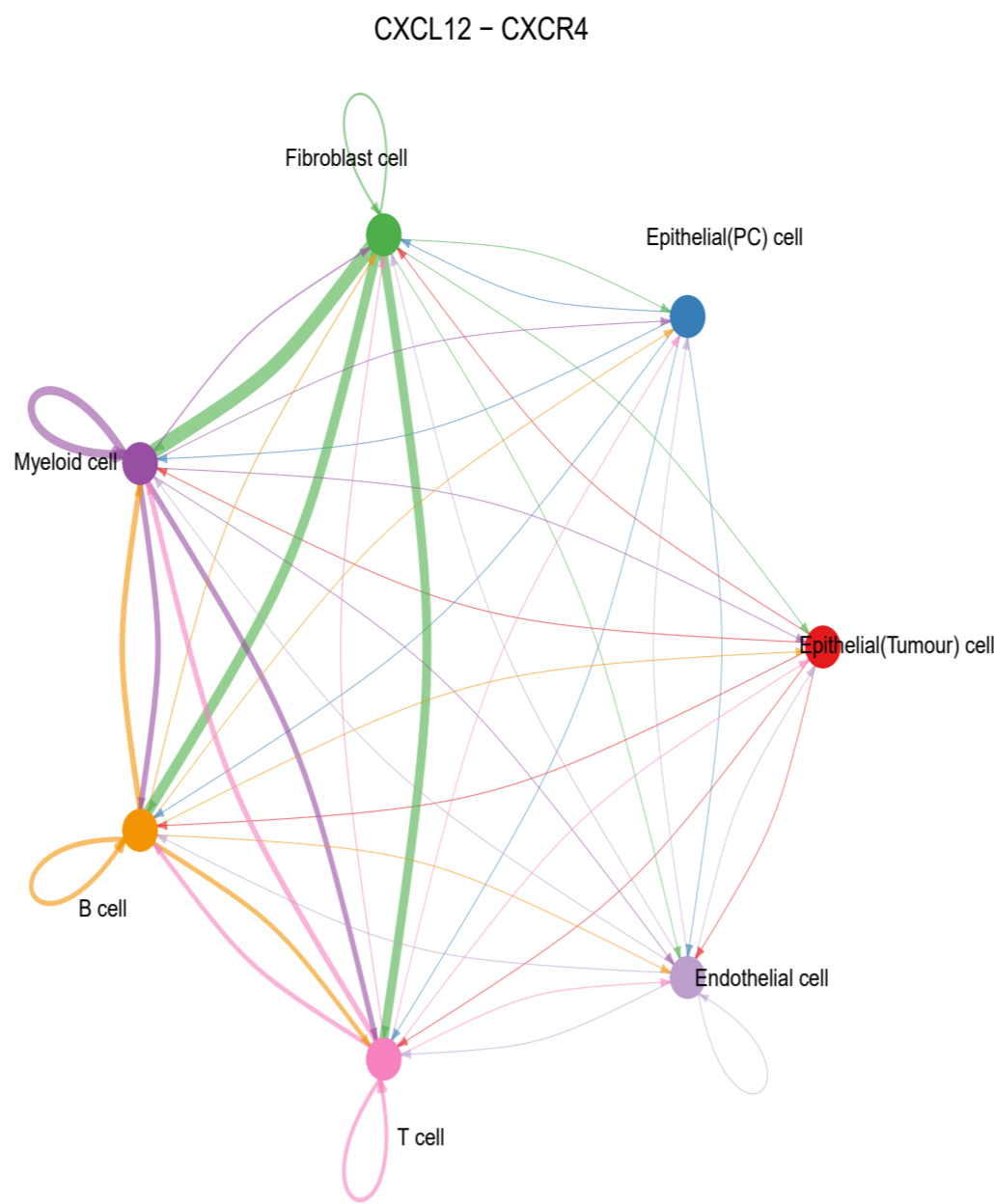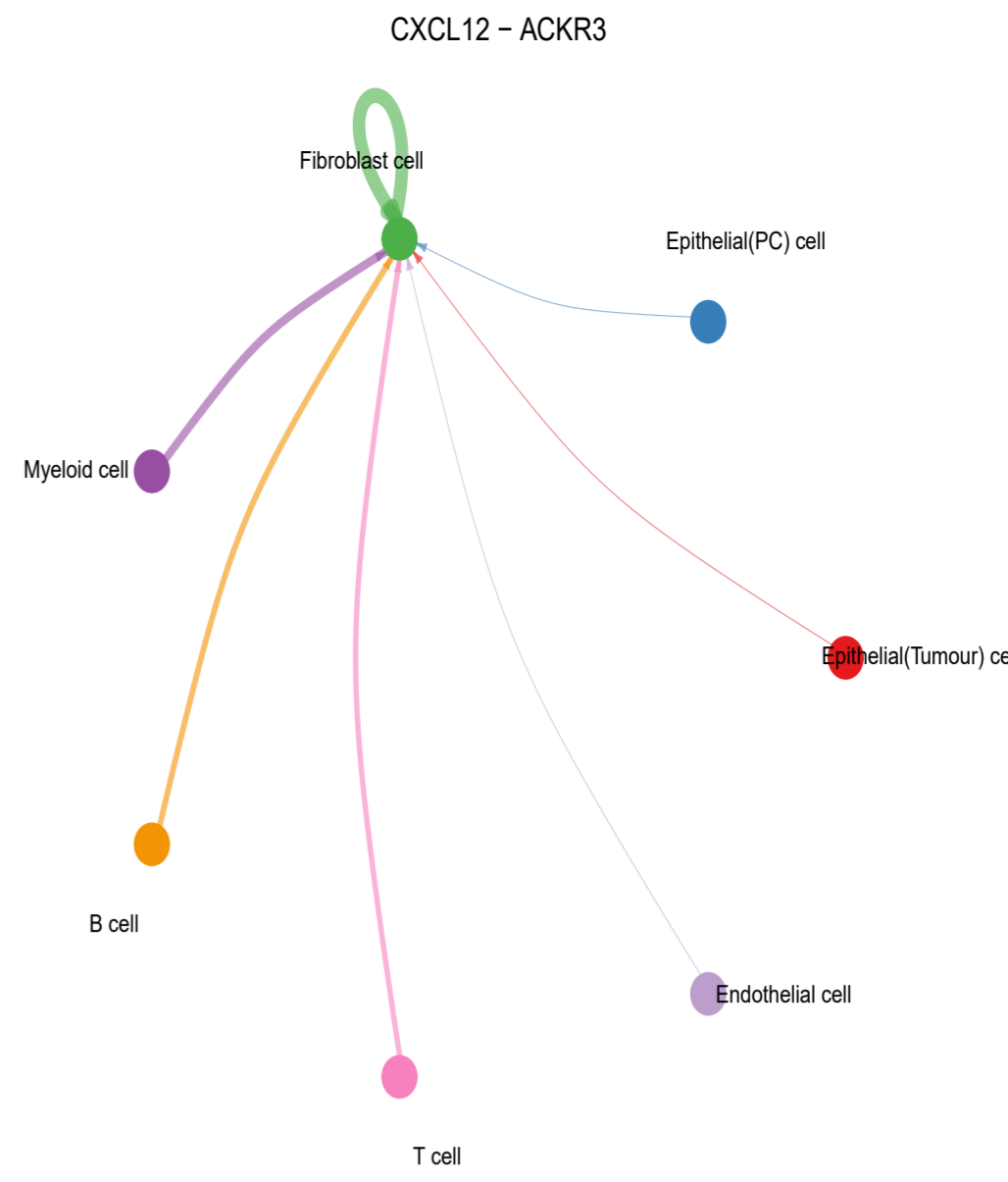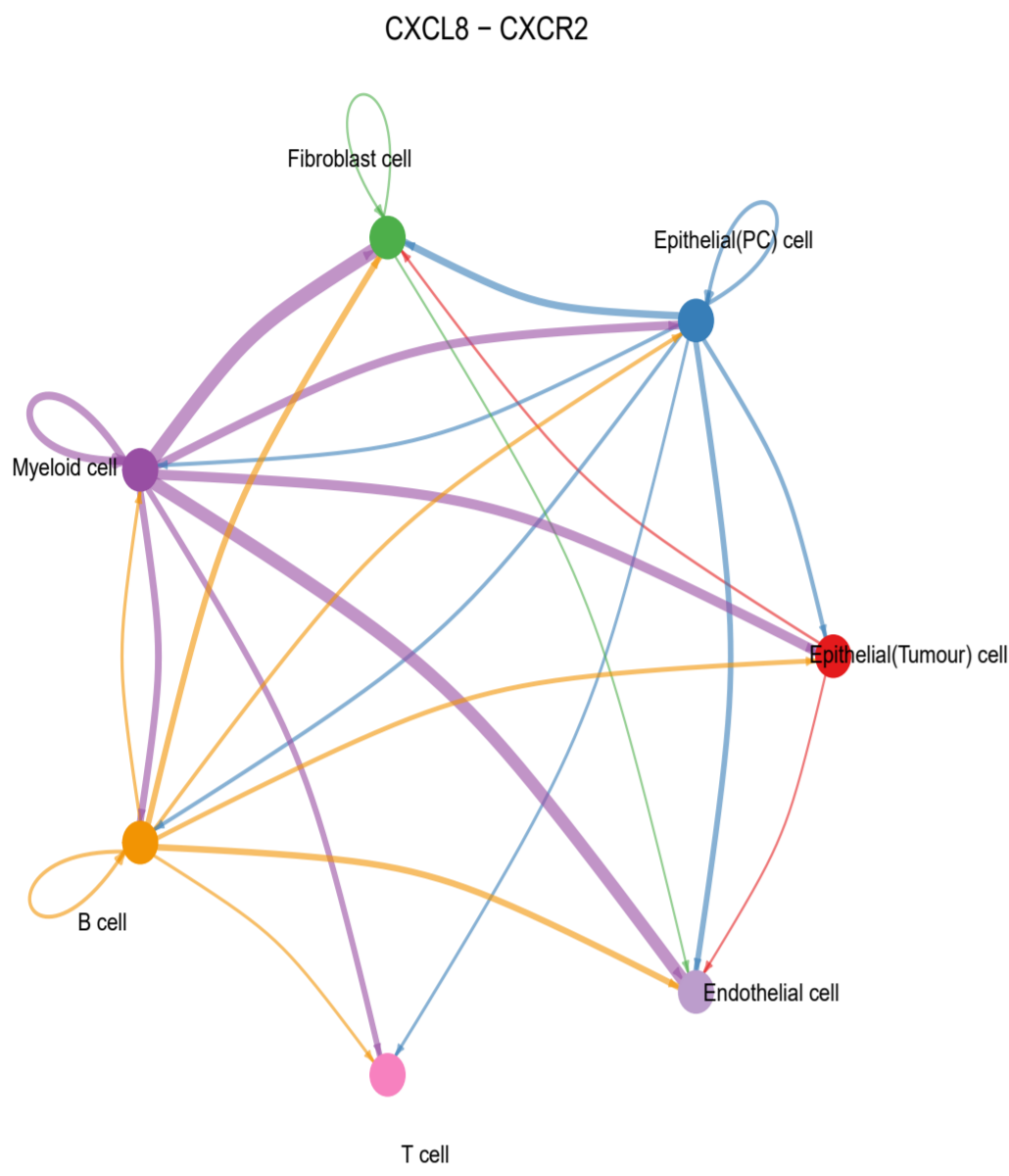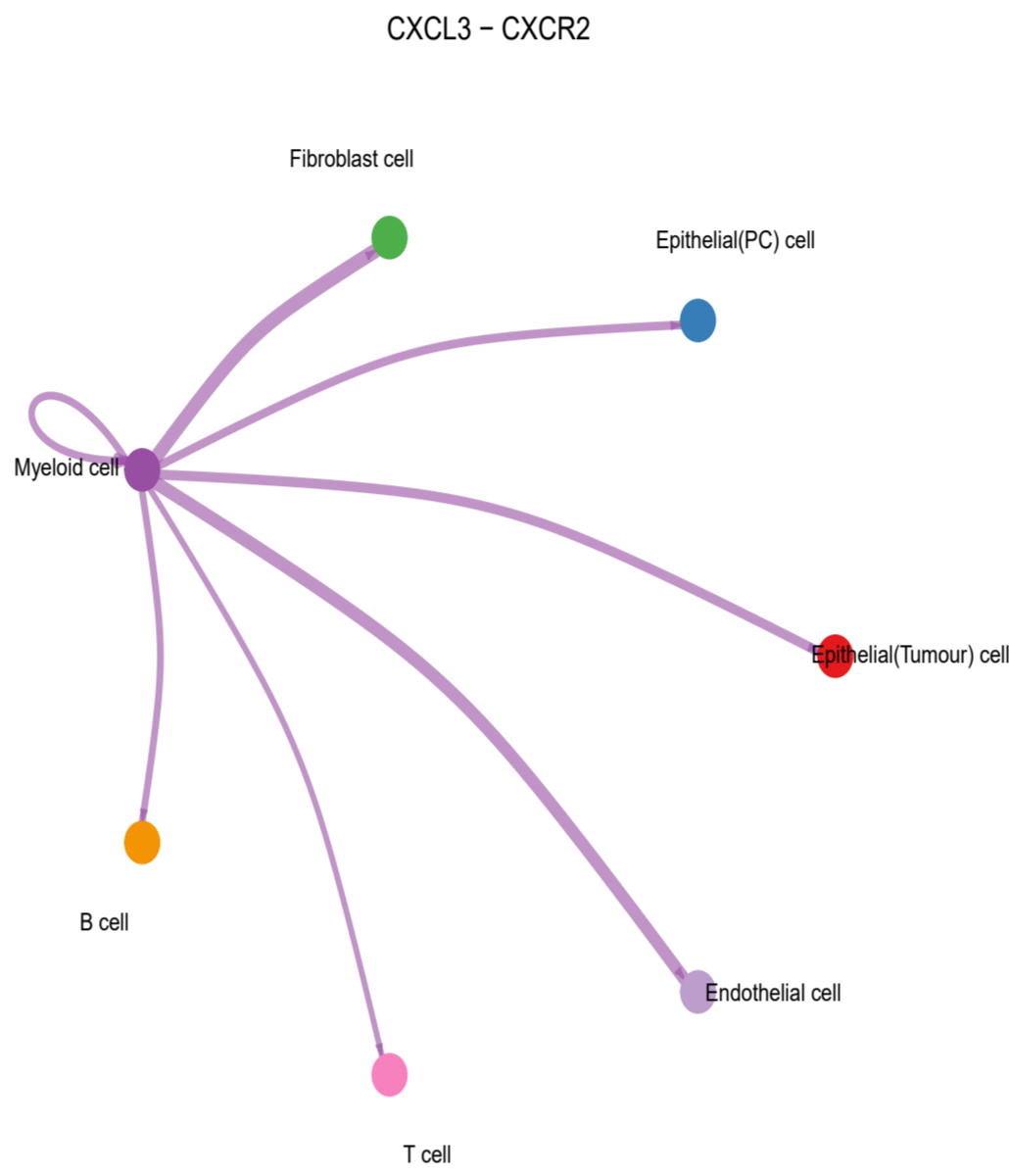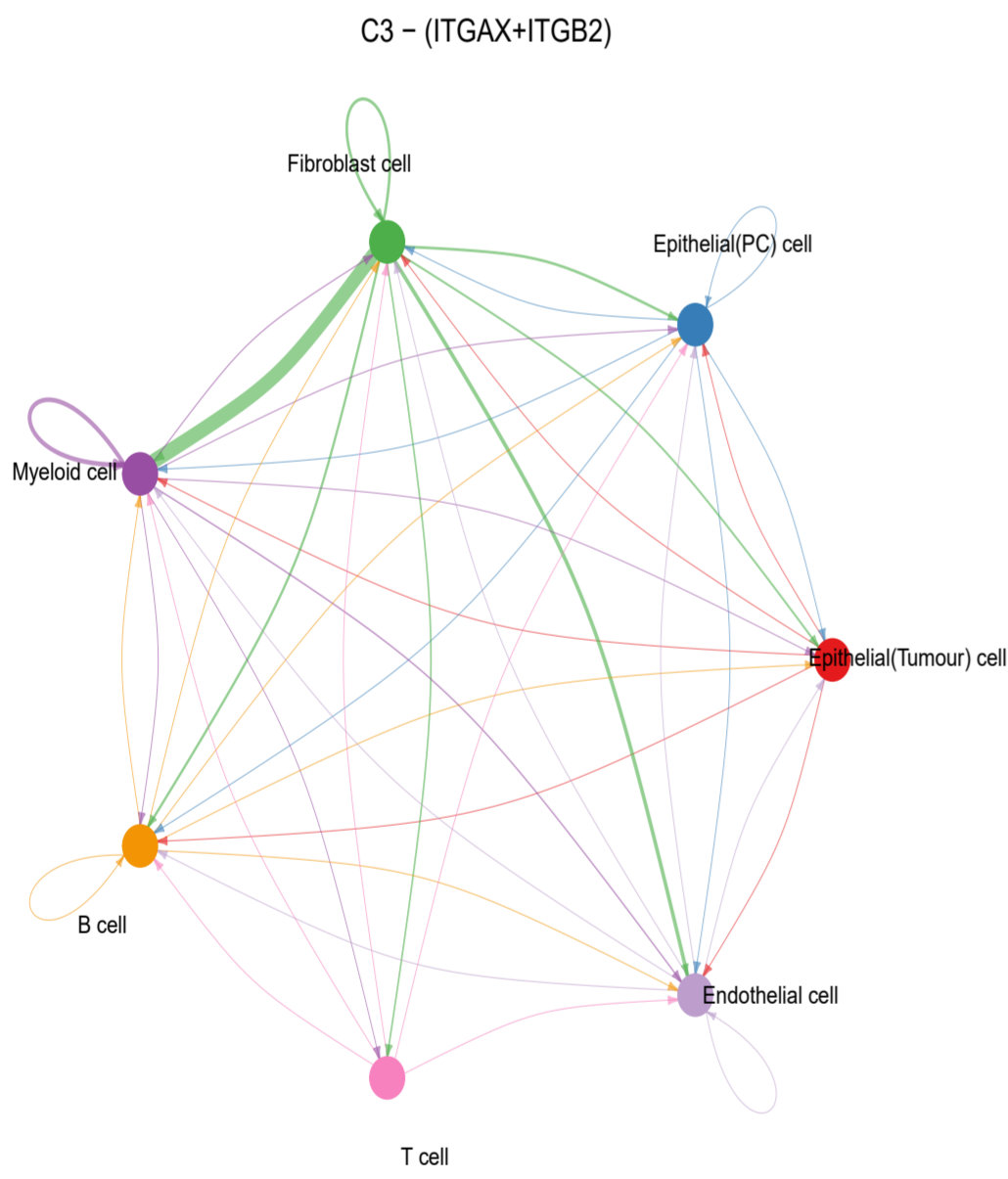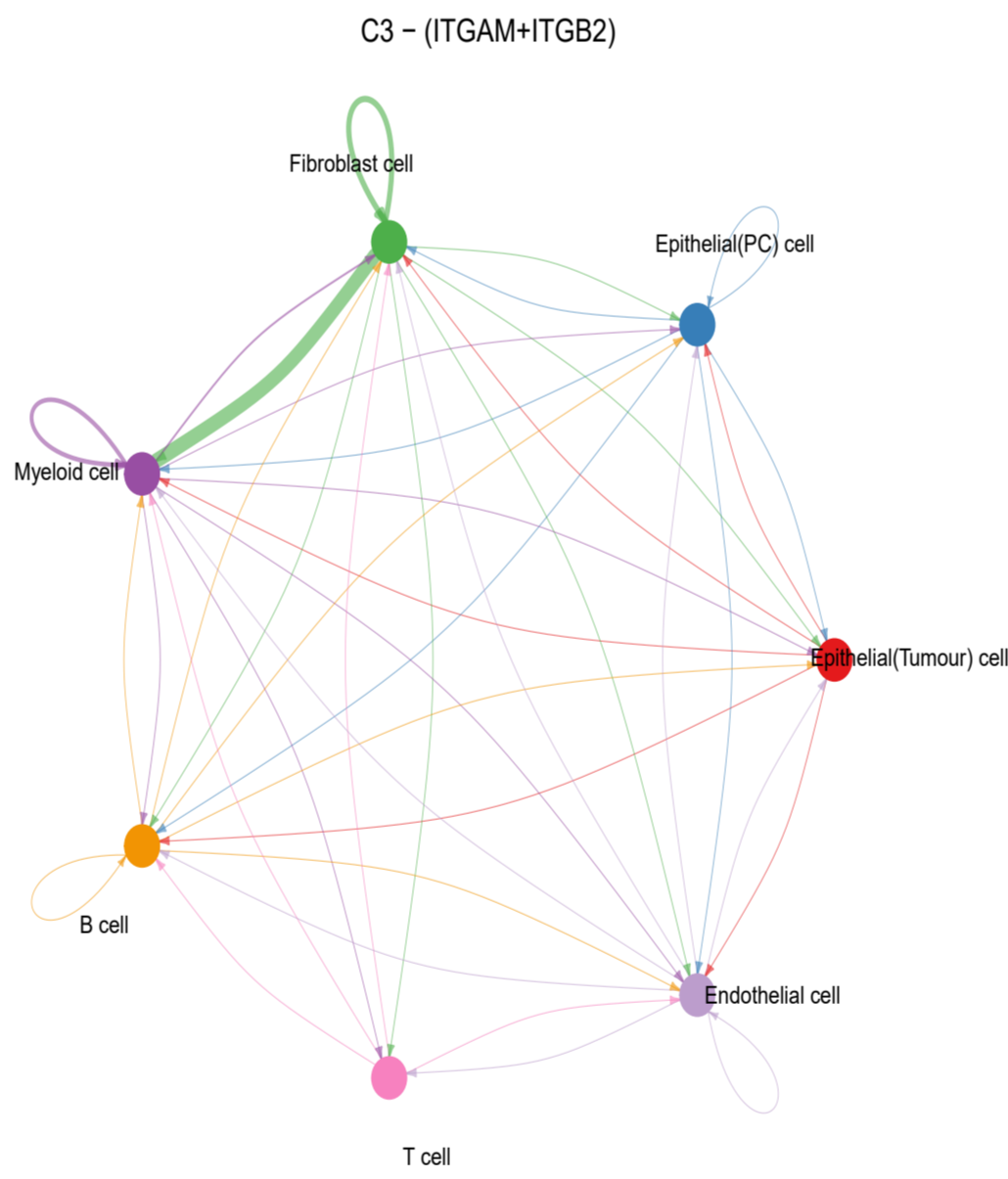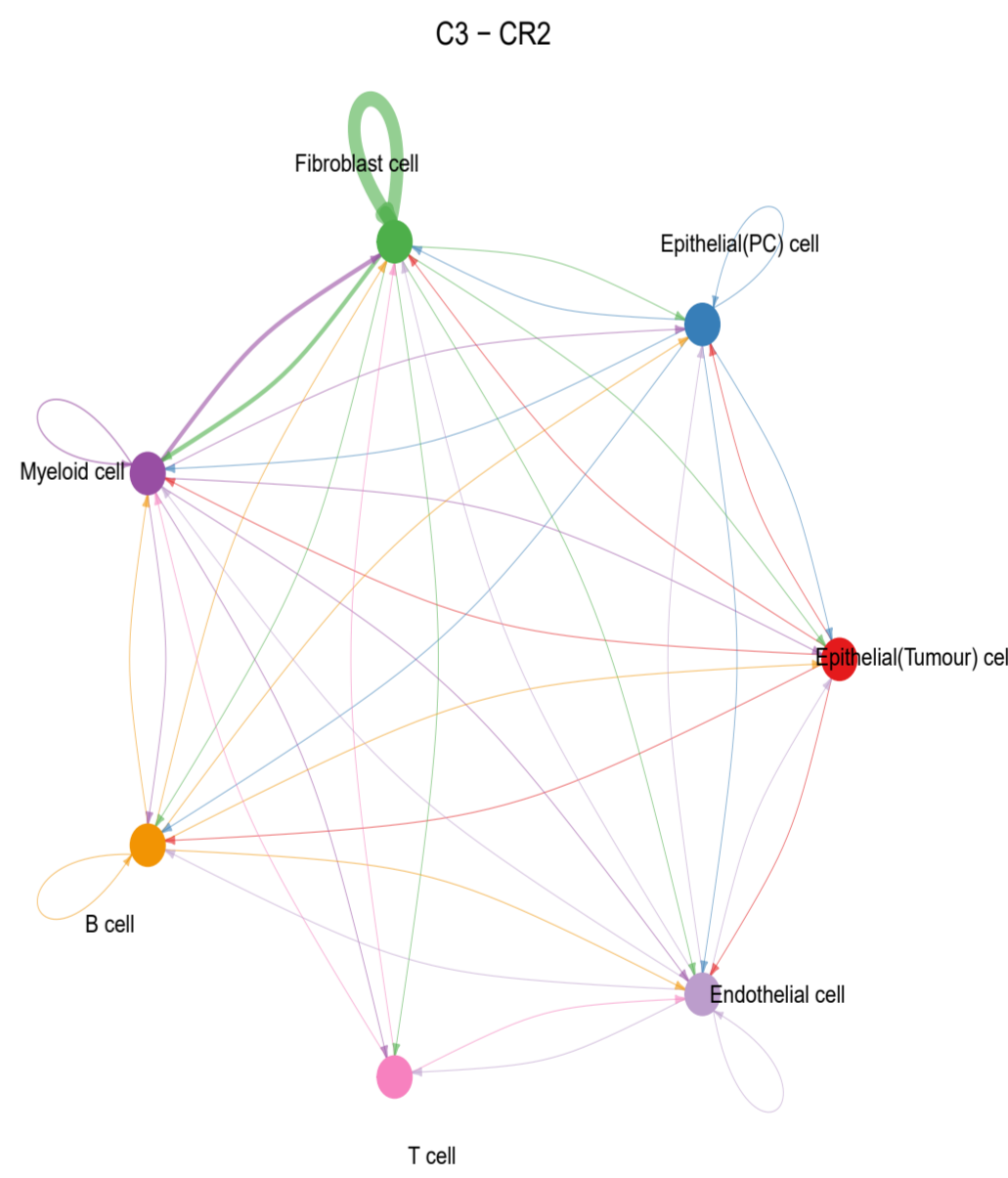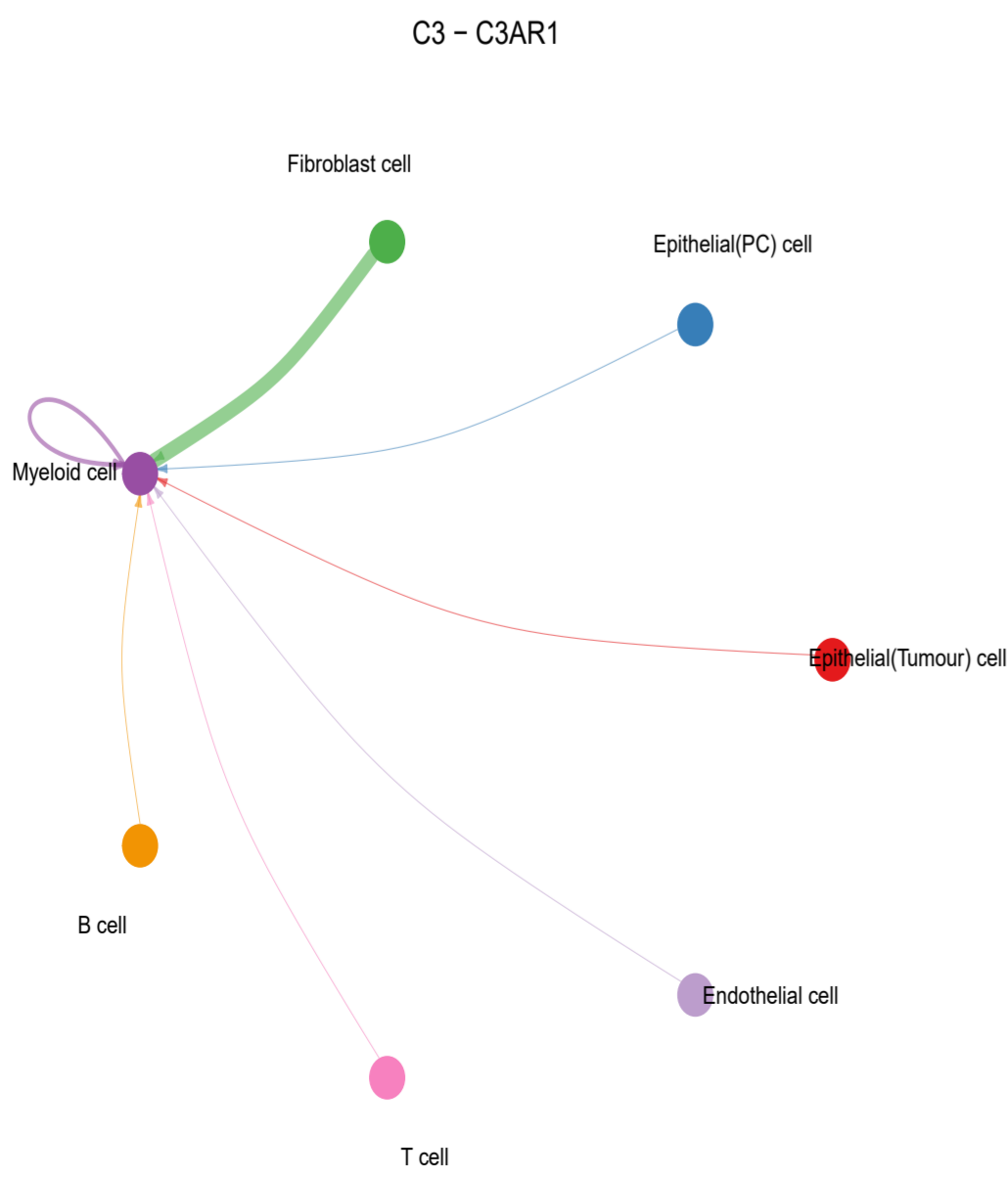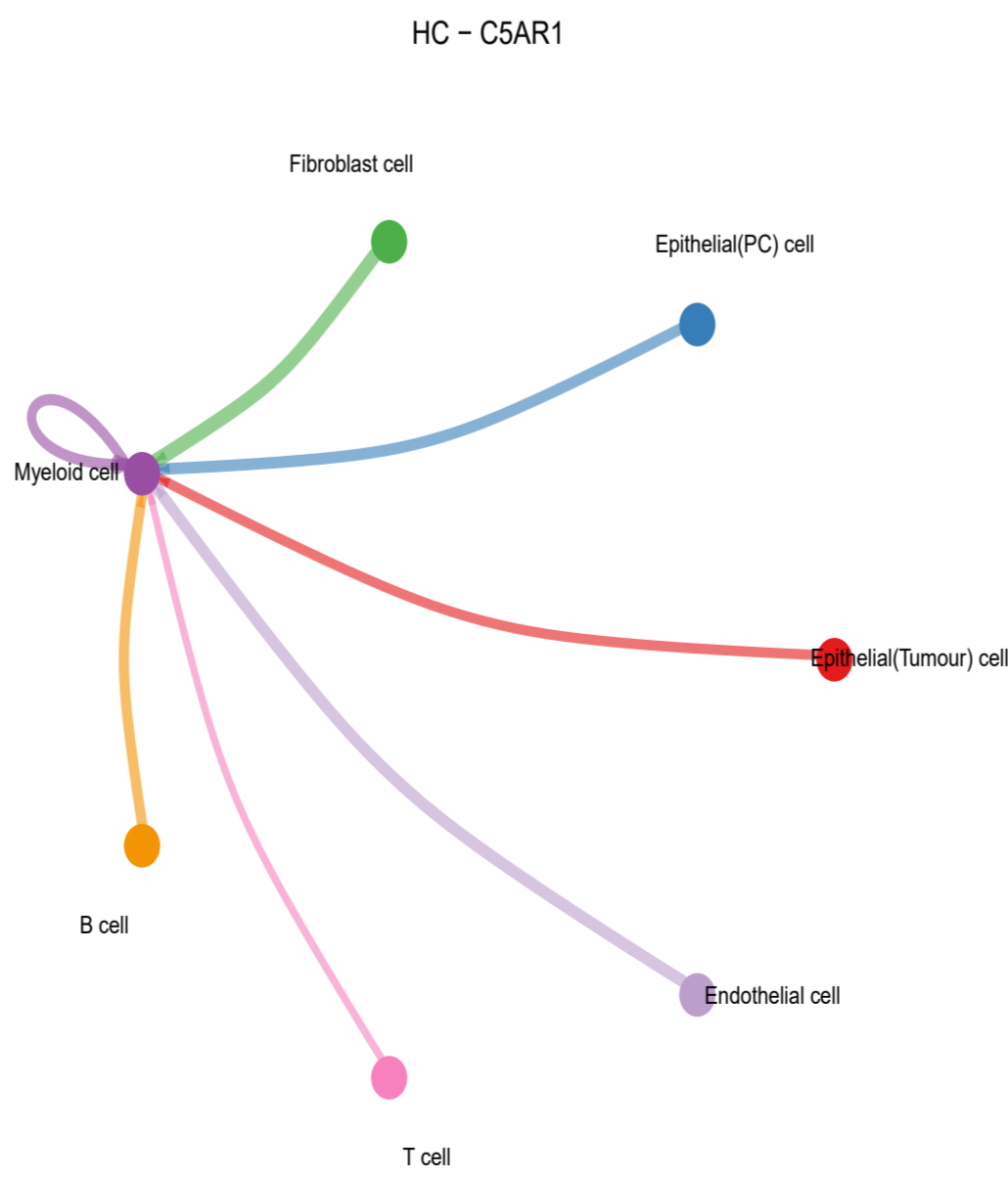

Supplement: Supplementary Figure 3 — Cell-cell communication analysis of the tumour microenvironment. Interactions between cell clusters in CXCL signaling pathway and Complement signaling pathway. [file Image_3.pdf]

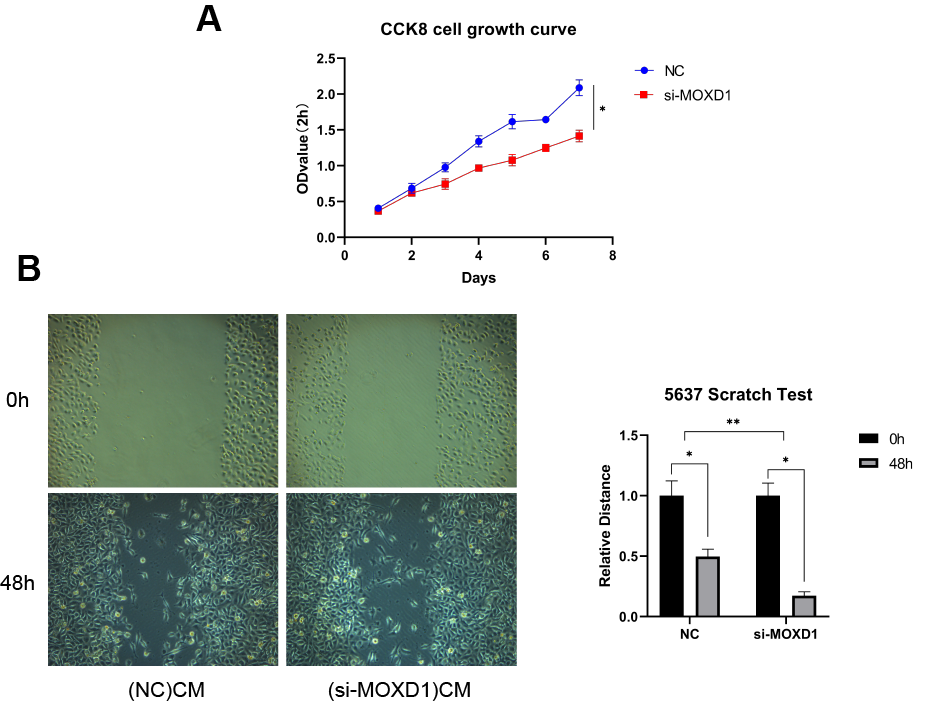

Supplement: Supplementary Figure 4 — Cellular function experiments in 5637 cell line (A) The CCK8 cell growth experiment was used to analyze the influence of stromal MOXD1 on the proliferation ability of 5637 cells. The results are presented as the mean optical density (OD) at 450 nm for triplicate wells two hours after the incubation. The results are presented as the mean ± SD of three independent experiments (* p < 0.05, * * p < 0.01, * * * p < 0.001). (B) The scratch wound assay determined that knock-down of MOXD1 in fibroblasts attenuated the migration ability of 5637 cells. The quantifications of cell migration were presented by the histogram. [file Image_4.png]
